# Single Extracellular VEsicle Nanoscopy-Universal Protocol (SEVEN-UP): Accessible Imaging Platform for Quantitative Characterization of Single Extracellular Vesicles

**DOI:** 10.1021/acs.analchem.4c04614

**Published:** 2025-01-13

**Authors:** Andras Saftics, Benjamin Purnell, Balint Beres, S. Thompson, Nan Jiang, Ima Ghaeli, Carinna Lima, Brian Armstrong, Kendall Van Keuren-Jensen, Tijana Jovanovic-Talisman

**Affiliations:** † Department of Cancer Biology and Molecular Medicine, Beckman Research Institute, City of Hope Comprehensive Cancer Center, Duarte, California 91010, United States; ‡ Department of Automation and Applied Informatics, Faculty of Electrical Engineering and Informatics, Budapest University of Technology and Economics, Budapest, H-1111, Hungary; § Light Microscopy/Digital Imaging Core, City of Hope Comprehensive Cancer Center, Duarte, California 91010, United States; ∥ Neurogenomics Division, Translational Genomics Research Institute, Phoenix, Arizona 85004, United States

## Abstract

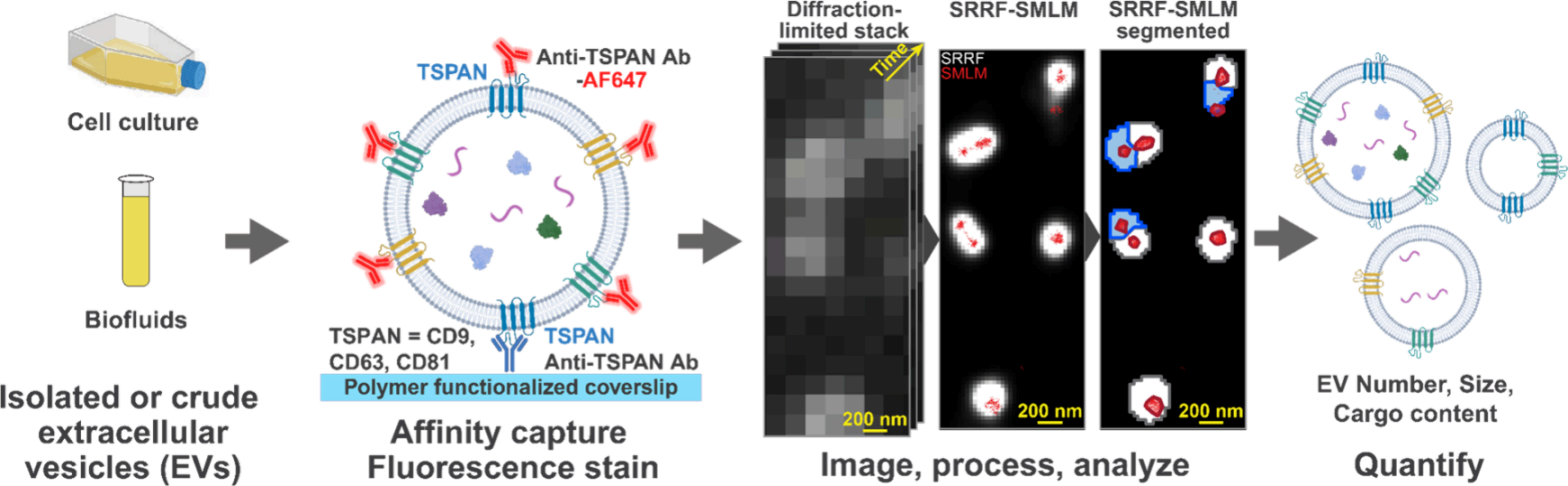

Extracellular vesicles (EVs), membrane-encapsulated nanoparticles shed from all cells, are tightly involved in critical cellular functions. Moreover, EVs have recently emerged as exciting therapeutic modalities, delivery vectors, and biomarker sources. However, EVs are difficult to characterize, because they are typically small and heterogeneous in size, origin, and molecular content. Recent advances in single EV methods have addressed some of these challenges by providing sensitive tools for assessing individual vesicles; one example is our recently developed Single Extracellular VEsicle Nanoscopy (SEVEN) approach. However, these tools are typically not universally available to the general research community, as they require highly specialized equipment. Here, we show how single EV studies may be democratized via a novel method that employs super-resolution radial fluctuations (SRRF) microscopy and advanced data analysis. SRRF is compatible with a wide range of microscopes and fluorophores. We herein quantified individual EVs by combining affinity isolation (analytical protocol based on SEVEN) with SRRF microscopy and new analysis algorithms supported by machine learning-based EV assessment. Using SEVEN, we first optimized the workflow and validated the data obtained on wide-field and total internal reflection fluorescence microscopes. We further demonstrated that our approach, which we call the SEVEN-Universal Protocol (SEVEN-UP), can robustly assess the number, size, and content of plasma and recombinant EVs. Finally, we used the platform to assess RNA in EVs from conditioned cell culture media. Using SYTO RNASelect dye, we found that 18% of EVs from HEK 293T cells appear to contain RNA; these EVs were significantly larger compared with the general EV population. Altogether, we developed an economical, multiparametric, single EV characterization approach for the research community.

## Introduction

All cells release membrane-encapsulated nanoparticles—extracellular vesicles (EVs)—that contain protein, lipid, nucleic acid, and metabolite cargo molecules.^1,2^ EVs are key orchestrators of intercellular communication; they are involved in both essential physiological processes and the pathogenesis of diverse diseases, such as cancer, neurodegenerative disorders, and diabetes. As exemplified in over 200 National Institutes of Health-listed clinical trials, EVs have also emerged as important biomarker candidates, therapeutic modalities, and delivery vehicles.^3−8^ However, the enhanced understanding of EVs in health and disease has been impeded by lack of accessible methodology to characterize specific EV subpopulations.^9,10^ The issue arises from three main challenges. 1) Within any given sample, the biophysical properties of EVs are highly heterogeneous. EVs can span a large range of sizes (30–10,000 nm) and densities (1.08–1.21 g/L).^11,12^ These wide ranges cause overlaps with other types of abundant biological nanoparticles (e.g., lipoproteins and protein aggregates^11,13,14^). 2) Few methods can characterize multiple parameters of individual EVs with high sensitivity and rigor; they typically require specialized reagents (e.g., fluorescent dyes) and equipment. 3) All cells release soluble components, extracellular particles, and EVs; biofluids contain complex mixtures of different particle types. Molecules located on the EV surface can be targeted for their isolation. For example, membrane proteins and motifs highly expressed on EV membranes can be used to enrich EV subpopulations. Tetraspanins (TSPANs) CD9, CD63, and CD81 are highly expressed on most EVs from different cell types.^15^ Selective antibodies (Abs) against these proteins can be used to capture TSPAN-enriched EVs (representing a “general” EV population). Affinity reagents against other molecules on the EV surface can also be used for EV capture. This approach can help select EV subpopulations that show cell type, tissue, or disease enrichment.

We have recently combined affinity capture of EVs with quantitative single molecule localization microscopy (qSMLM) to enable multiparametric assessment of individual EVs.^16^ This new method called Single Extracellular VEsicle Nanoscopy (SEVEN) has excellent localization precision, high capturing efficiency, and the ability to assess crude biofluids. However, SEVEN requires a specialized microscope and can only be used with a subset of photocontrollable fluorophores. Here we employed analytical protocols we developed for SEVEN with super-resolution radial fluctuations (SRRF) microscopy.^17,18^ SRRF enables rapid data acquisition by reconstructing images from a short diffraction-limited image sequence (a few seconds of imaging time). Reconstruction is performed by calculating radial and temporal fluorescence intensity fluctuations; behaviors of fluorophores correlate in time, but those of noise do not.^17,19−27^ Importantly, we developed robust algorithms to quantify the acquired data. In particular, we used k-means clustering^28^ of Laplacian image segments and machine learning (ML)-based classification for resolving EVs located in close vicinity. The platform was tested using total internal reflection fluorescence (TIRF) and wide-field (WF) microscopy. We call this platform the SEVEN-Universal Protocol (SEVEN-UP).

## Experimental Section

### Antibodies and Fluorescent Probes

As reported previously,^16^ anti-CD9, anti-CD63, and anti-CD81 antibodies (Abs) were used for both affinity capture (unlabeled Abs) and detection (Alexa Fluor 647 [AF647] labeled Abs) of EVs. We refer to the combination of these three as anti-TSPAN Abs. Photophysical characteristics of anti-TSPAN Abs-AF647 (fluorescent reporters) were determined using the surface assay for molecular isolation (SAMI).^29^ Briefly, anti-TSPAN Abs were covalently attached onto coverslips in a very low surface density, so a single Ab could be imaged in a diffraction-limited spot (we refer to these samples as SAMI surfaces). Using SAMI surfaces, we assessed 1) maximum dark time and the average number of localization per fluorescent reporter (α) for single molecule localization microscopy (SMLM);^16,29^ and 2) the average intensity integral per fluorescent reporter for super-resolution radial fluctuations (SRRF) microscopy (see SRRF^TIRF^Image Analysis for SAMI Surfaces (640 nm Channel)). Cell permeable SYTO RNASelect Green Fluorescent Cell Stain (Thermo Fisher Scientific Inc.; cat. no. S32703) with reported emission of 530 nm for RNA was used to stain EV-associated RNA molecules. Abs are listed in section S1.1 of Supplemental Methods.

### EV Samples

Three types of EV-containing sources were used in this study: (1) pooled human plasma, (2) enhanced green fluorescent protein (eGFP) expressing recombinant EVs, and (3) EVs isolated from conditioned HEK 293T cell culture media.

#### Pooled Human Plasma EV Samples

Pooled human plasma (blood derived) was obtained from Innovative Research, Inc. (Cat# IPLAWBK2E50 ML, LOT# 34553; Novi, MI, USA). The blood plasma was collected and processed according to the protocol of Innovative Research described before.^16^ Diluted crude plasma was directly used for the imaging experiments. We refer to pooled human plasma EVs as pEVs.

#### Recombinant EV Samples

Recombinant EV (rEV) reference material was obtained from MilliporeSigma (Cat# SAE0193–1VL).^30^ Lyophilized rEVs were reconstituted in 100 μL of ultrapure water according to the manufacturer’s protocol.

#### HEK 293T EV Samples

Culturing of human embryonic kidney (HEK) 293T cells, the isolation of HEK 293T EVs (hEVs) as well as staining of hEVs with SYTO RNASelect are described in section S1.2 of Supplemental Methods.

### EV Characterization

Following the Minimal Information for Studies of Extracellular Vesicles (MISEV) 2023 guidelines,^31^ SEC-isolated hEV samples were characterized using UV–vis spectroscopy, nanoparticle tracking analysis (NTA), transmission electron microscopy (TEM), and dot blot techniques according to previously described protocols.^16,32,33^ To determine the eGFP positivity ratio of rEVs, NTA measurements were performed using rEV samples diluted to 1:200 in PBS and acquiring NTA frame sequences both with and without a 500 nm filter engaged. A thorough characterization of SEC-isolated pEV and rEV samples is detailed in our previous publication.^16^

### Affinity-Capturing and Staining of EV Samples According to SEVEN Assay

Three types of EV samples (pEVs, rEVs, and hEVs) were processed according to the protocol based on the SEVEN assay.^16^ Briefly, clean, polymer coated glass coverslip surfaces were functionalized with anti-TSPAN Abs.^16^ For pEV samples, crude pooled human plasma was diluted in PBS to 1:50; for rEV samples, EVs were diluted in PBS to 1:200; for SEC-isolated hEV samples, hEVs were treated with RNase and SYTO RNASelect (or DMSO as control), purified with qEV single SEC column, and diluted in PBS to 1:2. 80 μL of EV-containing sample (supplemented with 0.025% (v/v) Tween 20) was incubated overnight at RT on a rocking shaker onto these functionalized coverslips. Coverslips were washed and stained with anti-TSPAN Abs-AF647 as reported before.^16^ pEV and rEV samples were fixed as previously described;^16^ no fixation was applied for hEV samples following the recommendation of the manufacturer’s protocol for staining with SYTO RNASelect.

### Image Acquisition

#### SRRF^TIRF^, SMLM, and SRRF^WF^ Image Acquisition

SRRF in total internal reflection fluorescence (TIRF) mode (SRRF^TIRF^) and SMLM imaging was performed on a Nikon Ti2 3D N-STORM super-resolution microscope (Nikon Instruments Inc.; Melville, NY, USA); microscope components are described elsewhere in detail.^16,33,34^ Both SRRF^TIRF^ and SMLM image acquisitions were performed with a TIRF illumination. SRRF in wide-field (WF) mode (SRRF^WF^) imaging was performed on a ZEISS Observer 7 microscope (Carl Zeiss Microscopy GmbH; Oberkochen, Germany) using a Colibri 7 LED system with 630- and 475-nm channels, XYZ piezo stage, fast filter wheels, 63×/1.2 W autocorr water immersion objective, and Hamamatsu Fusion BT sCMOS Mono camera. For additional information about image acquisition, see section S1.3 of Supplemental Methods.

### Image Reconstruction

Raw multiframe SRRF images were processed using the enhanced SRRF (eSRRF) protocol^18^ (see Figure S1). For additional details regarding pre- and post-processing of SRRF^TIRF^ and SRRF^WF^ images, see section S1.4 in Supplemental Methods. SMLM image reconstruction and generation of localization coordinate maps (i.e., SMLM images) was performed in the N-STORM Offline Analysis Module of the NIS-Elements software as previously described.^16^ The alignment of SRRF and SMLM images as well as images acquired in different channels are detailed in section S1.5 of Supplemental Methods.

### SMLM Image Analysis

Aligned SMLM images were analyzed with MATLAB code employing Voronoi tessellation-based clustering of localizations^35^ as described before.^16,32^ Optimized clustering conditions and threshold parameter values are listed in section S1.8 of Supplemental Methods.

### Correlative SRRF^TIRF^-SMLM Image Analysis (640-nm Channel)

**Figure 1:**
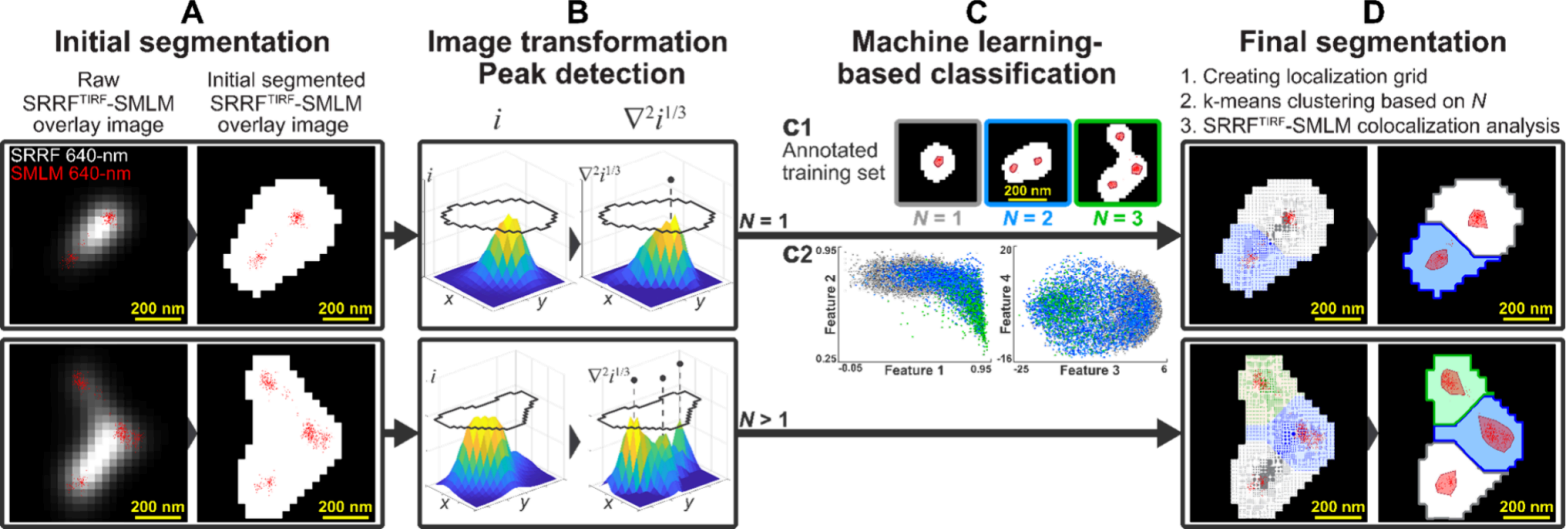
SRRF image analysis pipeline. We developed the SRRF image analysis pipeline based on correlative SRRF^TIRF^-SMLM images acquired using pEVs. A. Two SRRF^TIRF^ image sections (grayscale pixel-based images) are shown on the left, overlaid and aligned with their corresponding SMLM image sections (red localizations). The images show multiple SMLM-detected EVs located in close vicinity. First, we thresholded the SRRF^TIRF^ images to generate initial segments (binary white images on the right). The identified initial SRRF^TIRF^ segments were further analyzed to improve segmentation accuracy. B. We transformed the intensity signal of each initial segment (*i*, left) using cubic root and Laplacian transformation (∇^2^*i*^1/3^, right) and performed peak detection. C. When a single peak was detected in B (*N* = 1, top), we performed a machine learning-based ordinal classification to determine the number of EV components of initial segments based on 11 selected segment features. For classification, we used a support vector machine model with the training set of pEV SRRF^TIRF^-SMLM images. The training set contained selected features of initial segments annotated with the number of SMLM-detected EVs (ground truth) overlapping with their segment region (c1). EVs were identified as clusters of SMLM localizations detected by Voronoi tessellation-based clustering method. The feature space is represented by the 2D scatter plots where initial segments overlapping with one (*N* = 1, gray), two (*N* = 2, blue), or three (*N* = 3, green) EV components are shown (c2). When multiple peaks were detected (*N* > 1, bottom) in B, the number of EV components of initial segments was determined as the number of detected peaks. D. To further segment the initial segments based on *N*, we generated a grid of localizations over the foreground pixels scaled according to the ∇^2^*i*^1/3^ intensity values of pixels and performed k-means clustering on the grid localizations (left), identifying two (top) or three (bottom) distinct segments indicated in gray, blue, and green. The final SRRF^TIRF^ segmentations with outlined segments overlaid with SMLM-detected segments (outlined in red) are also shown (images on the right). We evaluated segmentation performance by comparing the number of predicted EV components with the true number of underlying EV components (i.e., SMLM-detected segments) by performing SRRF^TIRF^-SMLM colocalization analysis.

For the correlative SRRF^TIRF^-SMLM analysis, we split the images acquired on pEVs and rEVs into training and test sets. Training data sets of SRRF^TIRF^ and SMLM images consisted of 58 ROIs for pEV samples (77% of the entire set with *n* = 10 independent measurements) and 25 ROIs for rEV samples (76% of the entire set with *n* = 4 independent measurements). For validation, a test data set consisted of 17 ROIs for pEVs (23% of the entire set) and 8 ROIs for rEVs (24% of the entire set). SMLM images containing the determined localization cluster boundaries (list of polygon vertex coordinates) were used as the ground truth. Image analysis including SRRF image segmentation and the SRRF^TIRF^-SMLM colocalization analysis workflow is shown in Figure 1. The workflow included: a) Image binarization and preprocessing; b) Segment image transformation; c) Peak detection; d) Feature extraction; e) SRRF^TIRF^-SMLM colocalization of initial segments; f) Training classification model; g) Segmentation based on number of identified components; h) SRRF^TIRF^-SMLM colocalization of final segments and correlation analysis; (i) Validation of EV detection and segmentation. Detailed description is provided in section S1.9 of the Supplemental Methods.

### SRRF^WF^ Image Analysis (630-nm Channel)

The analysis of SRRF^WF^ images was performed by applying our developed segmentation method described in section S1.9 of Supplemental Methods steps a-d and g (training of the classification model and colocalization steps were not applied); in step a, SRRF^WF^ images were binarized using an optimized intensity threshold determined as 0.50% of maximum image intensity.

### SRRF^TIRF^ Image Analysis for SAMI Surfaces (640-nm Channel)

640-nm SRRF^TIRF^ images acquired on SAMI surfaces^16,29^ were binarized using adaptive thresholding. Following segmentation, the average value of the absolute segment intensity integral per ROI (i.e., the average SRRF^TIRF^ intensity integral of a single TSPAN Ab-AF647 fluorescent probe) was determined (⟨*J*_1,S_^SRRF,S^⟩). This value was subsequently used to calculate the average value of detected TSPAN molecule per EV per ROI (⟨*M*_EV_^SRRF,S^⟩) by dividing the absolute intensity integral of SRRF^TIRF^ segments for each EV (acquired for rEV and pEV samples; *M*_EV_^SRRF,S^*= J*_S_^SRRF^/⟨*J*_1,S_^SRRF,S^⟩) and averaging them across the ROI.

### Statistical Information and Graphics

Statistical analyses were performed in MATLAB and GraphPad Prism 10 (GraphPad Software, LLC; version 10.2.0; Boston, MA, USA). Details on statistics and information about graphics are provided in section S1.12 of Supplemental Methods.

## Results and Discussion

### Development and Validation of SEVEN-UP Using Plasma EVs

SEVEN analytical protocol^16^ was used to affinity isolate and stain EVs on glass coverslip surfaces. In particular, TSPAN-enriched EVs were captured on polymer-coated surfaces functionalized with anti-TSPAN Abs; EVs were subsequently stained with AF647-labeled anti-TSPAN Abs. Optimized imaging and analysis pipelines for SEVEN-UP were established using crude pooled human plasma EVs (pEV). For TSPAN-enriched pEVs, the same region of interest (ROI) was imaged in the 640-nm channel with SRRF in TIRF illumination mode (SRRF^TIRF^) followed by SMLM. Imaging parameters (most importantly laser power and acquisition time) for SRRF^TIRF^ were selected to maximize signal-to-noise ratio while imaging parameters for SMLM were selected to ensure that fluorophores blink efficiently with little overlap in the same frame (standard low acquisition times and high laser power settings). Because we acquired only 500 frames with low laser power in SRRF^TIRF^, no appreciable signal loss was observed in the SMLM following SRRF imaging. Optimized SRRF^TIRF^-SMLM acquisition procedure is detailed in section SRRF^TIRF^-SMLM image acquisition in Experimental Section. Raw multiframe SRRF^TIRF^ images were processed according to the established eSRRF method.^18^ Highlighted steps of the processing workflow with representative pEV images are shown in Figure S1. SMLM images were processed according to established protocols^16^ to generate localization maps.

We estimated the mean localization precision of SMLM images based on both photon counts^36^ and nearest neighbor analysis^37,38^ (for additional details, see section S1.6 in Supplemental Methods). Based on 77 pEV ROIs, we estimated the average localization precision to be in the range of 7–8 nm (Figure S2A). Performing Fourier ring correlation (FRC) analysis^38−40^ we also estimated the resolution of SMLM, SRRF^TIRF^, and SRRF^WF^ images (related methods are described in section S1.6 and S1.7 of Supplemental Methods). Since the FRC-based resolution estimation requires the analysis of structured image regions,^38^ as an approximation, we evaluated images with the highest detected EV density. As shown for a representative SMLM image in Figure S2B, we estimated a resolution of 28 nm based on the FRC curve calculated using MATLAB codes published by Martens et al.^38^ For SRRF^TIRF^ images we estimated minimum image resolutions based on FRC maps generated by the NanoJ-SQUIRREL ImageJ2 (Fiji) plugin.^40^ The FRC maps of representative SRRF^TIRF^ images (Figure S2C, left) indicated that a best resolution was ∼70 nm. We also show excellent signal-to-background ratio (SBR) for pEVs imaged using SRRF^TIRF^, Figure S2D-E.

To optimize segmentation of SRRF^TIRF^ images and to achieve optimal EV detection accuracy (relative to SMLM which served as ground truth), processed SRRF^TIRF^ and SMLM images were overlaid. Our image analysis process is shown in Figure 1. SMLM localization maps were clustered and EVs were identified using optimized Voronoi tessellation-based clustering method^16^ (also see section SMLM image analysis in Experimental Section and S1.8 in Supplemental Methods). In our SRRF^TIRF^ segmentation approach (described Experimental Section and in section S1.9 of Supplemental Methods in detail), we first applied an initial thresholding to generate binary images and labeled initial segments (Figure 1A). To improve segmentation accuracy and detect single EVs, the identified SRRF^TIRF^ initial segments were further analyzed. For each detected initial segment, we generated a Laplacian of the cubic-root-transformed image (∇^2^*i*^1/3^) to improve peak separation (Figure 1B); the cubic root transformation reduced the intensity difference between strong and weak peaks for single segments composed of multiple peaks. Using a peak detection algorithm, we identified the number of peaks within the initial segments. For initial segments with multiple identified peaks, the final number of segment components was determined based on the number of detected peaks (bottom line). For initial segments with a single identified peak, we applied another round of analysis using a machine learning (ML)-based ordinal classification approach to decide if a segment contains multiple components (top line). To reduce oversegmentation, we did not consider those initial segments for further segmentation which had low variation in their intensity (for further details, see Figure S3). Toward the ML-based approach, we developed a support vector machine with a Gaussian kernel classification model using the training image set where the initial segments were annotated with the number of overlapping SMLM-detected EVs (i.e., number of true segment components). Overlaps were determined by performing SRRF^TIRF^-SMLM colocalization analysis. We trained the classification model using the annotated segments and selected segment features related to morphology and intensity as well as histogram of oriented gradients (HOG) (Figure 1C). Next, we generated a localization grid over the foreground pixels of those segments that had multiple identified components; the grid localization density was linearly scaled according to pixel intensity in the ∇^2^*i*^1/3^ segment image. Based on the identified number of components, we conducted a final segmentation of multicomponent initial segments performing k-means clustering on the generated localizations. Finally, we assessed segmentation performance by identifying overlaps between the detected SRRF^TIRF^ segments and SMLM clusters in a final SRRF^TIRF^-SMLM colocalization analysis (Figure 1D).

**Figure 2:**
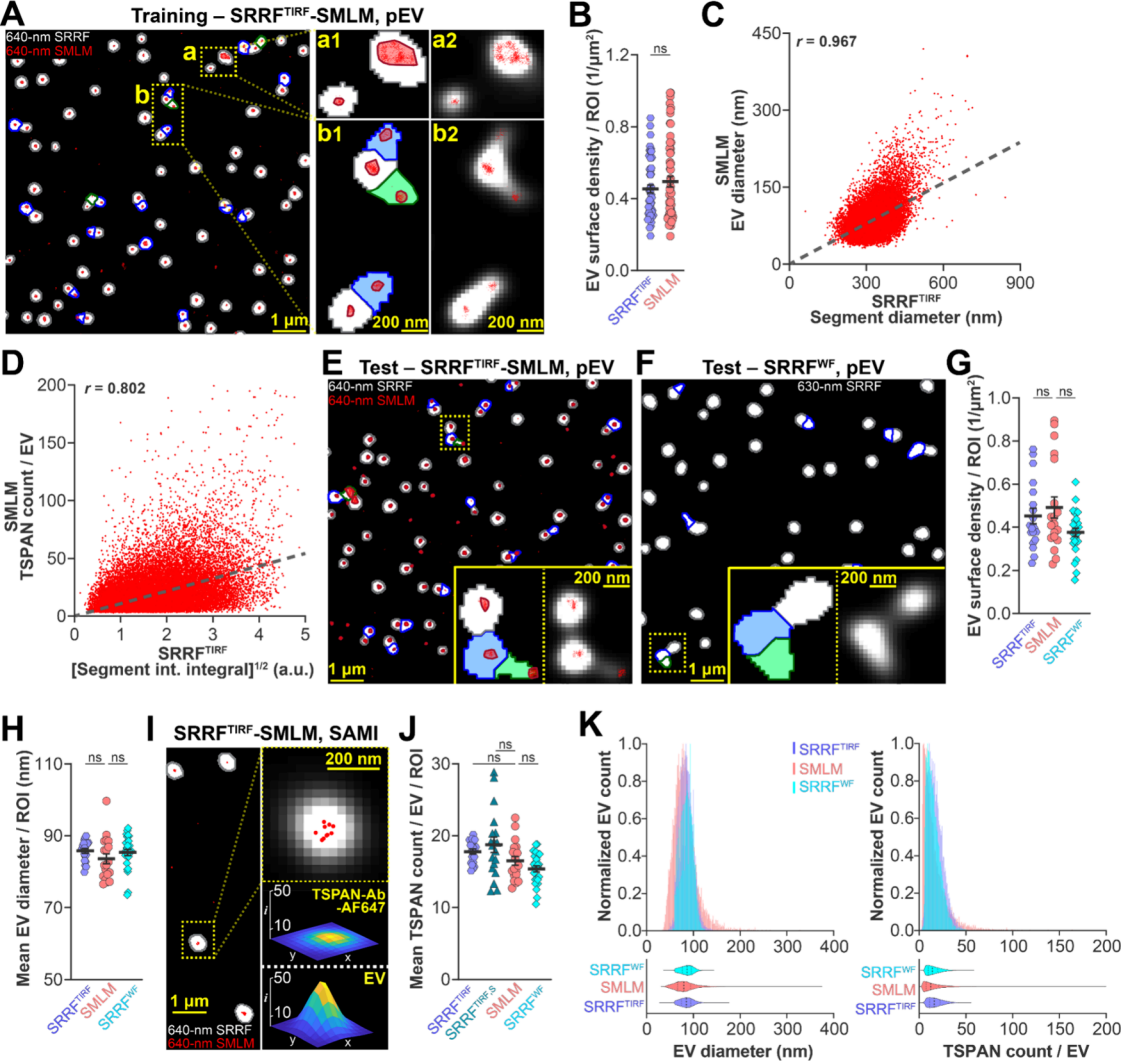
| Validation of SEVEN-UP using TSPAN-enriched and TSPAN detected crude pooled human plasma EVs. Representative training set ROI of a segmented 640-nm channel SRRF^TIRF^ image (segments outlined in gray, blue, and green) overlaid with corresponding 640-nm channel SMLM image (red localization map). Two enlarged segmented areas (a1, b1) and their corresponding raw images without segmentation (a2, b2) are shown. B. Comparison of SRRF^TIRF^- and SMLM-based EV surface density per ROI obtained by analyzing images of the training set to validate segmentation performance (*n* = 10 independent measurements, 58 ROIs). C. Scatter plot of SRRF^TIRF^-based segment diameter correlated with the SMLM-based EV diameter for colocalized single pEVs. D. Scatter plot of SRRF^TIRF^-based square root of normalized intensity integral of identified segments correlated with the SMLM-based detected number of TSPAN molecules for colocalized single pEVs. For C and D, linear regression models (gray dashed line) representing the relationship of SRRF^TIRF^ and SMLM data with corresponding correlation coefficient (*r*) values are shown (*n* = 10 independent measurements, 58 ROIs). E. Representative ROI of a segmented 640-nm channel SRRF^TIRF^ image from the test set overlaid with its corresponding SMLM image. The inset image shows an enlarged section of the ROI (as indicated by the dashed box) with corresponding raw image on the right. F. Representative ROI of a segmented 630-nm channel SRRF^WF^ image selected from the test set. The inset image shows an enlarged section of the ROI (as indicated by the dashed box) with corresponding raw image on the right. G-H. Comparison of SRRF^TIRF^- and SRRF^WF^-based EV surface density per ROI (G) and mean EV diameter per ROI (H) with the SMLM-determined EV characteristics. The regression model determined in graph C was used to calculate EV diameter from the segmented SRRF^TIRF^ and SRRF^WF^ images (*n* = 10 independent measurements, 19 ROIs for SRRF^TIRF^; *n* = 10 independent measurements, 19 ROIs for SMLM; *n* = 4 independent measurements, 27 ROIs for SRRF^WF^). I. Characterization of the fluorescent signal of single fluorescent probe molecules detected by SRRF^TIRF^ on a representative SAMI surface. The right panel on top shows the signal on a SRRF^TIRF^ image segment identified as a single fluorophore, validated by SMLM (red localizations). The right panel on the bottom shows SRRF^TIRF^ image segment of a detected single EV for comparison. J. Comparison of SRRF^WF^- and SRRF^TIRF^-based mean TSPAN count per EV per ROI with the SMLM-determined EV characteristic (*n* = 10 independent measurements, 19 ROIs for SRRF^TIRF^; *n* = 10 independent measurements, 19 ROIs for SMLM; *n* = 4 independent measurements, 27 ROIs for SRRF^WF^). The regression model determined in graph D was used to calculate the number of TSPAN molecules per EV from segmented SRRF^TIRF^ and SRRF^WF^ images. In addition to the regression-based method, the number of detected TSPAN molecules per EV per ROI was also determined using the SRRF^TIRF^ signal of a single fluorophore measured on SAMI surfaces (SRRF^TIRF,S^; *n* = 10 independent measurements, 19 ROIs). Error bars represent mean ± SEM; ns indicates that the difference between corresponding data sets was determined as statistically not significant (*p* ≥ 0.05). K. Histograms and related violin plots representing the distribution of EV diameter (left) and detected TSPAN count per EV (right) of individual pEVs determined by SRRF^TIRF^, SMLM, and SRRF^WF^. Data were from the test sets (*n* = 10 independent measurements, 19 ROIs for SRRF^TIRF^; *n* = 10 independent measurements, 19 ROIs for SMLM; *n* = 4 independent measurements, 27 ROIs for SRRF^WF^).

Representative SRRF^TIRF^ and SMLM image of pEVs is shown in Figure 2A. SRRF^TIRF^ pEV segments outlined in gray, blue, and green with SMLM segments outlined in red are shown in the left panel, while zoomed-in areas are shown in the middle panel (a1, b1); for the same zoomed-in areas, reconstructed SRRF image in white pixels with SMLM localizations in red dots are shown in the right panel (a2, b2). Well segmented structures with good overlap between SRRF^TIRF^ and SMLM were apparent. For training, we used an image set that contained 58 ROIs (each 1,678 μm^2^). pEV images had a relatively high surface density of EVs (number of detected EVs per unit surface area); the number of SRRF segments that overlapped with multiple SMLM-detected EVs was on average 11%. We detected 48,140 pEVs in SMLM and 44,166 in SRRF. Importantly, there was no significant difference in pEV surface density between SMLM and SRRF analysis (Figure 2B). Next, for the subset of 37,692 pEVs in the training set (in this subset, each SRRF^TIRF^-detected EV was colocalized with a single SMLM EV to avoid any ambiguity), the SMLM-determined size (EV diameter) was correlated with the SRRF^TIRF^-determined size (segment diameter). We found a strong positive correlation with a correlation coefficient of *r* = 0.967, Figure 2C. Next, we modeled the detected molecule count using linear regression based on the intensity integral. We identified, using a power-transform-based optimization, that the model achieves better predictive performance when a square root operation is applied on the input feature. For the same 37,692 pEVs in the training set, we then correlated the detected molecular TSPAN content (TSPAN molecule count per EV) and square root of normalized intensity integral of segment ([segment intensity integral]^1/2^) of identified single pEVs. We found a strong positive correlation with correlation coefficient of *r* = 0.802, Figure 2D. To validate our protocol, we used the SRRF^TIRF^-SMLM data set containing 19 ROIs (15,680 SMLM-detected pEVs). Representative test image regions shown in Figure 2E show similar features observed in the training set (Figure 2A).

We also acquired SRRF images of pEVs using wide-field illumination (SRRF^WF^; optimized image acquisition and processing conditions are detailed in the section SRRF^WF^ Image Analysis (630-nm Channel) and applied our segmentation protocol. A representative test image region (Figure 2F) shows well segmented pEVs. Of note, while we used dSTORM imaging buffer in all cases (necessary for efficient fluorophore switching in SMLM), there was no significant difference in the detected number of pEVs between dSTORM and PBS buffers (Figure S4). The FRC maps of representative SRRF^WF^ images (Figure S2C, right) indicated that a best resolution was ∼70 nm. While SBR was lower for SRRF^WF^ compared to SRRF^TIRF^ (as expected due to different illumination mode), values were still in good range, Figure S2D-E. This was in part due to the relatively high number of detected tetraspanins on EVs.

We validated the detected pEV numbers for the SRRF^TIRF^ and SRRF^WF^ test sets. There was no significant difference in pEV surface densities between SMLM and the two SRRF test sets, Figure 2G. We obtained 85% overlap between SRRF^TIRF^ and SMLM. While 8% of SRRF segments did not have an overlapping SMLM cluster, only 1% of SRRF-identified pEVs did not show detected localizations in SMLM. This is expected because we defined SMLM cluster when 2.5 or more TSPAN molecules were detected to reduce signal originating from other cellular components and noise. Thus, we had a very low false positive rate. We also assessed the accuracy of SRRF^TIRF^ image segmentation, Figure S5. Here we evaluated whether the number of overlapping SMLM-detected pEVs for an initial SRRF^TIRF^ segment (true EV count) was accurately predicted by our trained method (predicted EV count). The confusion matrix shows the percentage of segments within a specific class (value on the top left corner of each cell) and the absolute value of segments within a specific cell. 97.5% of segments with 1 overlapping pEV and 73.9% of segments with 2 overlapping pEVs were accurately segmented as 1-component and 2-component segments, respectively. Although the segmentation accuracy dropped for segments with 3 or more overlapping pEVs, it is important to note that the majority of these segments were correctly identified as multicomponent segments (e.g., 71.9% of segments with 3 overlapping pEVs were identified as 2-component segments, and 95.6% of segments with 4 overlapping pEVs were identified as 2- or 3-component segments). In addition, as the absolute counts show, only a small fraction of segments (1.4%) had 3 or more overlapping pEVs. Of note, due to the rarity of segments with more than 4 components, we trained the classification model to classify initial segments with up to three underlying EV components. Still, as the count of 3-component initial segment samples was low, the model was skewed toward predicting lower component counts. This could be improved with different augmentation methods which could perform the equalization of class samples in the training set.

Using the linear regression model (Figure 2C), next we determined the SRRF-based EV diameter and calculated the mean for each analyzed ROI. The mean pEV diameter shows good agreement between the SMLM and two SRRF test sets acquired on TIRF and WF microscopes, Figure 2H.

We next performed an independent calibration to obtain molecular content information from the SRRF^TIRF^ images. We measured the mean SRRF^TIRF^ signal of individual fluorescent reporters (anti-TSPAN Abs-AF647) using the SAMI surfaces that enables imaging of individual fluorescent reporters^16,29^ (Figure 2I). The ratio of the absolute intensity integral of an EV SRRF segment (example shown in bottom right section of Figure 2I) and the mean of absolute intensity integral for individual fluorescent reporter yielded TSPAN count per EV for SRRF images (see section SRRF Image Analysis for SAMI Surfaces (640-nm Channel) in the Experimental Section). For test ROIs we compared the obtained TSPAN molecular counts using this approach (SRRF^TIRF,S^) with values obtained with SMLM; we also compared TSPAN molecular counts obtained using the linear regression model from Figure 2D (SRRF^TIRF^ and SRRF^WF^) with SMLM. We found that the difference between the mean values of TSPAN contents for each of SRRF^TIRF^, SRRF^TIRF,S^, and SRRF^WF^ and the SMLM data were not significant (Figure 2J). Thus, using the developed calibrations, SEVEN-UP can report detected molecular TSPAN content information in addition to size and EV counts.

Histograms and corresponding violin plots of size and TSPAN molecular count distributions for individual EVs indicated good agreement between the imaging methods (Figure 2K). For single pEVs colocalized in the overlaid SRRF^TIRF^-SMLM images of the test set, we further correlated calibrated SRRF^TIRF^-based diameter and TSPAN content with the SMLM-determined diameter and TSPAN content. As shown in Figure S6A, we observed strong positive correlation for both size (*r* = 0.97) and TSPAN count (*r* = 0.80). Although we found that proper characterization of large EVs and EVs with very high TSPAN content is limited, it is worth noting that they represent only a small group of the total detected EV population (98% of pEVs had a size below 150 nm, and 99% of pEVs had a TSPAN count below 75). The poor correlation in this range may also be attributed to our training set, which had a low number of EVs with diameter over 150 nm as well as TSPAN count over 75. A training set with a broader size and TSPAN count distribution and better coverage in the higher ranges would likely improve the correlation.

### Validation of SEVEN-UP Using rEVs

**Figure 3:**
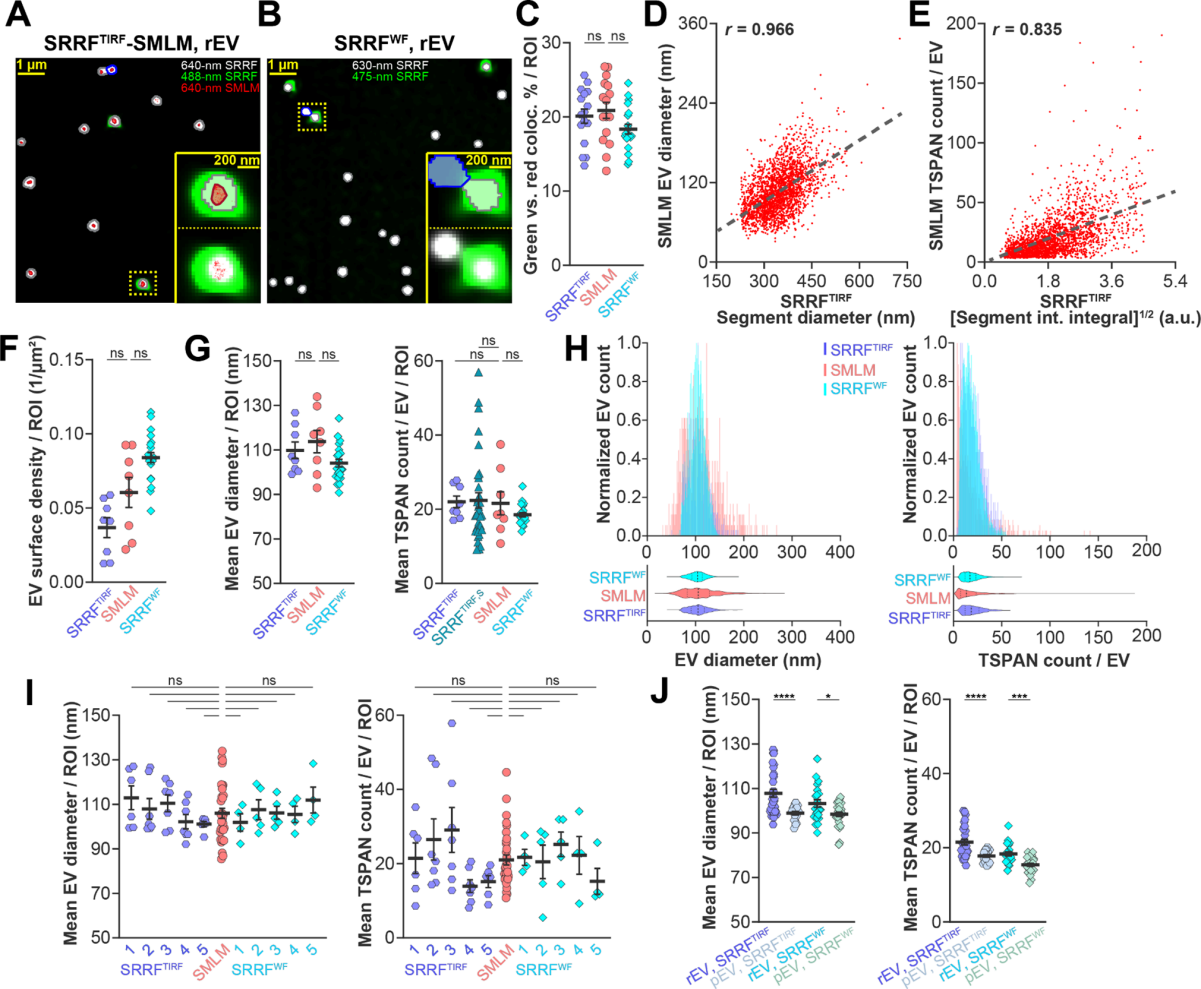
Validation of SEVEN-UP using eGFP containing TSPAN-enriched and TSPAN detected recombinant EVs. A. Representative ROI of a segmented 640-nm channel SRRF^TIRF^ image (segments outlined in gray and blue) and raw 488-nm channel SRRF^TIRF^ image (green) overlaid with its corresponding 640-nm channel SMLM image (red localization map). The inset image shows an enlarged section of the ROI (as indicated by the dashed box) with corresponding raw image on the bottom; GFP signal is shown in green color (488-nm channel). B. Representative ROI of a segmented 630-nm channel and raw 475-nm channel SRRF^WF^ image as well as its enlarged section (inset, top) with corresponding raw image (inset, bottom); GFP signal is shown in green color. C. Fraction of green vs red channel colocalization per ROI determined by SRRF^TIRF^, SMLM, and SRRF^WF^ (*n* = 4 independent measurements, 16 ROIs for SRRF^TIRF^; *n* = 4, 16 ROIs for SMLM; *n* = 4 independent measurements, 20 ROIs for SRRF^WF^). D. Scatter plot of SRRF^TIRF^-based segment diameter correlated with the SMLM-based EV diameter for colocalized single rEVs. E. Scatter plot of SRRF^TIRF^-based square root of normalized intensity integral of identified segments correlated with the SMLM-based detected number of TSPAN molecules for colocalized single rEVs. For D and E, linear regression models (gray dashed line) representing the relationship of SRRF^TIRF^ and SMLM with corresponding correlation coefficient (*r*) values are shown (*n* = 4 independent measurements, 25 ROIs). F-G. Comparison of SRRF^TIRF^- and SRRF^WF^-based EV surface density per ROI, mean EV diameter per ROI, and mean TSPAN count per EV per ROI with the SMLM-determined EV characteristics (*n* = 4 independent measurements, 8 ROIs for SRRF^TIRF^; *n* = 4 independent measurements, 8 ROIs for SMLM; *n* = 4 independent measurements, 22 ROIs for SRRF^WF^). The regression models determined in graph D and E were used to calculate EV diameter and TSPAN content from segmented SRRF^TIRF^ and SRRF^WF^ images. In addition to the regression-based method, the number of detected TSPAN molecules per EV was also determined based on the SRRF^TIRF^ signal of a single fluorophore measured on SAMI surfaces (SRRF^TIRF,S^). H. Histograms and related violin plots representing the distribution of EV diameter (left) and detected TSPAN count per EV (right) of individual rEVs determined by SRRF^TIRF^, SMLM, and SRRF^WF^. Data were from the test sets (rEVs: *n* = 4 independent measurements, 8 ROIs for SRRF^TIRF^; *n* = 4 independent measurements, 8 ROIs for SMLM; *n* = 4 independent measurements, 22 ROIs for SRRF^WF^). I. 5-fold cross-validated calibration of SRRF-based mean segment diameter per ROI and mean intensity integral per ROI of rEVs with SMLM-determined mean diameter and mean TSPAN content of rEVs. The determined SRRF^TIRF^-based (left-hand side of graphs) and SRRF^WF^-based (right-hand side of graphs) EV characteristics in each cross-validation test set (1–5) were compared against the SMLM-based EV characteristics (center) (*n* = 4 independent measurements, 33 ROIs for SRRF^TIRF^; *n* = 4 independent measurements, 33 ROIs for SMLM; *n* = 4 independent measurements, 22 ROIs for SRRF^WF^). J. The difference in EV diameter and TSPAN content between pEVs and rEVs was successfully detected by SRRF^TIRF^ and SRRF^WF^ using the calibration based on rEVs (rEVs: *n* = 4 independent measurements, 33 ROIs for SRRF^TIRF^; *n* = 4 independent measurements, 22 ROIs for SRRF^WF^; pEVs: *n* = 10 independent measurements, 19 ROIs for SRRF^TIRF^; *n* = 4 independent measurements, 27 ROIs for SRRF^WF^). Error bars represent mean ± SEM; ns indicates *p* ≥ 0.05, * indicates *p* < 0.05; *** indicates *p* < 0.001; **** indicates *p* < 0.0001.

In addition to pEVs, we also used commercially available recombinant reference EVs (rEVs)^30^ to validate SEVEN-UP. A fraction of rEVs contains eGFP and thus can be used to confirm EV detection by their inherent fluorescence, allowing us to further validate SRRF-based EV detection in the 488-nm SRRF^TIRF^ and 475-nm SRRF^WF^ channel. We affinity isolated rEVs on anti-TSPAN Abs-immobilized coverslips and stained the captured rEVs with anti-TSPAN Abs-AF647. We imaged the surfaces using 1) correlative SRRF^TIRF^-SMLM in 640-nm channel to detect TSPANs followed by SRRF^TIRF^ in 488-nm channel to detect GFP; or 2) SRRF^WF^ in 630-nm channel to detect TSPANs followed by SRRF^WF^ in 475-nm channel to detect GFP (GFP is not compatible with SMLM imaging). Based on 33 ROIs, we estimated on average a 7–9 nm localization precision of SMLM images, Figure S2A. As before, in the 640-nm channel, good overlap between SMLM segments (outlined in red) and SRRF^TIRF^ segments (outlined in gray and blue) was evident (Figure 3A). Additionally, for GFP-positive rEVs, clear overlaps between 640/630- and 488-/475-nm channels in SRRF^TIRF^ (Figure 3A) and SRRF^WF^ (Figure 3B) were evident. We determined the GFP-positivity as the ratio of EVs colocalized in the green (488- or 475-nm) and red (640- or 630-nm) channels relative to all red channel-detected EVs (further details about the colocalization analysis can be found in section S1.10 of Supplemental Methods). As shown in Figure 3C, both SRRF^TIRF^ and SRRF^WF^ imagings provided similar values. Furthermore, the measured 18–21% mean GFP-positivity was in good agreement with independent fluorescence nanoparticle tracking analysis (NTA) measurements which resulted in 28% mean GFP-positivity, Figure S7. This demonstrates the ability of SEVEN-UP to identify the abundance of a distinct EV population in a multicolor imaging setting.

We performed optimized segmentation on a training image set of SRRF^TIRF^-SMLM rEV images (25 ROIs, containing 3,023 SMLM-detected rEVs in the 640-nm channel). We correlated the SMLM-detected diameter and molecular TSPAN counts with the SRRF^TIRF^-detected segment diameter and square root of the normalized intensity integral data, respectively. For this purpose, we used a subset of 2,435 EVs (as for pEVs, SRRF segments that overlapped with multiple SMLM-detected EVs were not included). We determined a strong positive correlation in both cases (*r* = 0.966 and 0.835, respectively, Figure 3D-E). The segmentation was applied on the test image set (8 ROIs, 812 SMLM-detected rEVs). We found no significant difference between the SRRF and SMLM in EV surface densities (Figure 3F). We obtained 87% overlap between SRRF^TIRF^ and SMLM. While 15% of SRRF segments did not have an overlapping SMLM cluster, only 1% of SRRF-identified rEVs did not show detected localizations in SMLM. Again, an SMLM cluster was defined when 2.5 or more TSPAN molecules were detected. While it is possible that because of the thresholding a small fraction of EVs with very low tetraspanin content was missed by both SMLM and SRRF, the model still performed well.

Using the established calibrations, we determined the mean EV size and mean TSPAN content per ROI from SRRF images and compared these data with the SMLM-derived EV size and TSPAN content. For TSPAN content, we also applied the SAMI-based independent calibration (diagrammed in Figure 2I). As shown in Figure 3G, we obtained excellent overlap between SRRF^TIRF^- and SRRF^WF^-determined EV sizes and TSPAN contents and corresponding SMLM data. SMLM- and SRRF-obtained histograms and related violin plots of EV size and TSPAN molecular count distributions for individual EVs showed good overlap (Figure 3H). Similar to pEVs, we evaluated the single EV characterization power of SEVEN-UP using rEVs (Figure S6B); 98% of the detected total rEV population had a size below 200 nm and 99% had a TSPAN count below 100 molecules/EV. A higher correlation in TSPAN content for rEVs (*r* = 0.83) compared to pEVs (*r* = 0.80) may be attributed to the higher TSPAN content of rEVs (better coverage of high TSPAN count EVs for calibration). TSPAN content in rEVs also led to excellent SBR for SRRF^TIRF^ and SRRF^WF^ (Figure S2D-E). Consequently, calibrations with markers that have a very low EV abundance could be challenging for SEVEN-UP.

As rEVs are commercially available and reproducible reference EVs with a stable size range and TSPAN content, our goal was to use rEVs as a standard to calibrate the SRRF signal, eliminating the need for SMLM acquisition. As described in detail in section S1.11 of Supplemental Methods, we determined calibration sensitivity values (slope of calibration curve determined from linear regression) for both the size and TSPAN content of rEVs; we divided the mean diameter per ROI of SRRF segments by the mean rEV size (106 nm based on 33 SMLM ROIs) and mean square root of intensity integral per ROI of SRRF segments by the mean TSPAN content (21 molecules/EV based on 33 SMLM ROIs). First, we applied a 5-fold cross-validation-based approach by randomly dividing the SRRF^TIRF^ and SRRF^WF^ ROIs into training and test sets five times to determine calibration sensitivities (from the training set) and validate the determined calibration sensitivities (on test sets) by comparing the calculated SRRF-based mean EV size per ROI and mean TSPAN content per ROI with related SMLM-derived data. As shown in Figure 3I, none of the validation test sets for SRRF^TIRF^-based (left-hand side of graphs) and SRRF^WF^-based (right-hand side of graphs) size and TSPAN content showed a significant difference compared to SMLM.

Applying rEV-based calibration sensitivities for size and TSPAN content (based on 33 ROIs), we calculated the size and TSPAN content of rEVs and pEVs from SRRF images, Figure 3J. For both SRRF^TIRF^ and SRRF^WF^, we detected significant difference between the size and TSPAN content of rEVs and pEVs. Note that although the absolute values in pEVs were skewed toward higher values, SEVEN-UP was able to detect the size and TSPAN content difference for rEVs and pEVs (22 nm and 5 molecules/EV according to SMLM).

As rEVs are reproducible, commercially available standards, our strategy can enable the accessibility of this technique to laboratories without an SMLM capable microscope that would still be necessary for initial calibration (albeit with some limitations regarding the accuracy of absolute values, especially when EVs of interest have largely different average size distributions). As additional recombinant EVs are becoming commercially available, (e.g., from Vesiculab Ltd.), calibration using multiple standards can increase the accuracy of the approach.

Here we focused on developing a methodology using TSPAN-enriched EVs and counting detected TSPAN molecules. Importantly, SEVEN is an easily customizable method that enables the capture and detection of desired molecules on EVs. For example, we have previously detected a rare, IGF1R-enriched EV subpopulation in patients with pancreatic cancer.^16^ Thus, this approach could be easily tailored to address a wide range of questions in EV biology and biotechnology. Moreover, since the approach is compatible with a range of fluorophores, it can be used to investigate genetically encoded EV constructs (e.g., exomap1 mouse that contains humanized CD81 tagged with mNeonGreen in EVs from specific cell types^41^) and fluorescent dyes that stain various cargo molecules.

### Quantification of EV-Associated RNA Content Using SEVEN-UP

We isolated EVs from HEK 293T cell culture media (hEVs) by using size exclusion chromatography (SEC). UV–vis spectrophotometry measurements showed that fractions F1–F5 had low total protein concentration (Figure S8A); thus, as before,^16^ we used combined fractions F1–F5 for our experiments. We characterized EVs according to the Minimal Information for Studying Extracellular Vesicles (MISEV) 2023 guidelines.^10^ Using dot blots, we confirmed that canonical EV markers (TSPANs as well as luminal EV protein syntenin) had high expression, while cytochrome C, which is typically not associated with EVs, had low expression (Figure S8B). Using nanoparticle tracking analysis (NTA), we assessed hEV concentration and size distribution obtaining (5.3 ± 0.4) × 10^9^ particle/mL and mean size of 164 ± 1 nm (Figure S8C). Transmission electron microscopy (TEM) demonstrated intact hEV morphology (Figure S8D).

**Figure 4:**
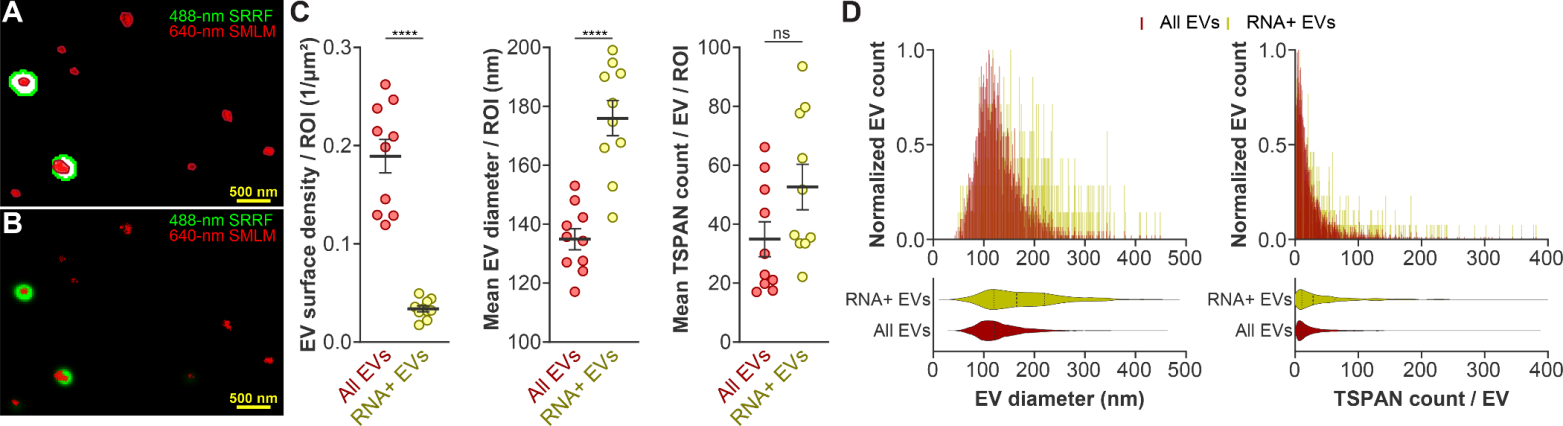
| Quantification of EV-associated RNA content of TSPAN-enriched HEK 293T EVs stained with anti-TSPAN Abs-AF647 and SYTO RNASelect dye. A. Segmented 488-nm channel SRRF^TIRF^ image of hEVs (outlined in green) was overlaid with its corresponding 640-nm channel SMLM image which was clustered using Voronoi tessellation (outlined in red). B. Representative 488-nm channel SRRF^TIRF^ raw image in green overlaid with 640-nm channel SMLM localizations in red. C. EV surface density per ROI, mean EV diameter per ROI, and mean detected TSPAN molecule count per EV per ROI for all TSPAN-enriched EVs and RNA-positive, TSPAN-enriched EVs. Error bars represent mean ± SEM (*n* = 3 independent measurements, 10 ROIs); ns indicates *p* ≥ 0.05; **** indicates *p* < 0.0001. D. Histograms and related violin plots representing the distribution of SMLM-detected EV diameter (left) and detected TSPAN count per EV (right) for individual hEVs in the TSPAN-enriched population (All EVs, red) and RNA-positive subpopulations (yellow). *n* = 3 independent measurements, 10 ROIs.

We treated hEVs with RNase to remove any RNA that may be associated with the outer EV membrane and subsequently used membrane permeable SYTO RNASelect dye to stain luminal RNA. While DNA content has only a minor interference effect when staining RNA content with SYTO RNASelect stain,^42^ the approach may result in small amount of nonspecific staining. Importantly, using bead-based flow cytometry, it was previously demonstrated that the SYTO RNASelect dye could be successfully applied to determine the RNA content of EVs.^43^ Here, SYTO RNASelect-treated hEVs were separated from excess reagents using SEC (see the protocol in section S1.2 of Supplemental Methods), captured on anti-TSPAN Abs-coated coverslips, and stained with anti-TSPAN Abs-AF647. We have combined SMLM to image TSPANs using anti-TSPAN Abs-AF647 as fluorescent reporters and SRRF^TIRF^ to image RNA using SYTO RNASelect as a fluorescent reporter. Of note, while SYTO RNASelect can photoswitch, in our hands (using ∼108 mW power of 488-nm laser at the fiber), the efficiency of blinking was not satisfactory; thus, SRRF allowed us to quantitatively assess RNA-positive EVs. Segmented (Figure 4A) and unsegmented (Figure 4B) representative images show EVs (red) and RNA (green); a fraction of EVs had RNA. We quantified the number of TSPAN-enriched EVs and those that contain RNA; based on SYTO RNASelect staining, 18% of TSPAN-enriched EVs were RNA positive (Figure 4C, left). In the control experiment, when SYTO RNASelect was not included, a minimal fraction of EVs had green signal (Figure S9). We next investigated the properties of RNA-positive EVs. Interestingly, our data suggest that these EVs are significantly larger (Figure 4C, middle); their TSPAN content was slightly but not significantly increased (Figure 4C, right). Histograms of sizes and TSPAN content confirm the unique properties of RNA-positive EVs (Figure 4D).

While we detected a low fraction of RNA positive EVs (18%), these results are in line with recent finding that ∼10% of small EVs are enriched in RNA.^44^ Of note, our threshold for SYTO RNASelect was stringent with a low false positive signal (5.7%). Given that our data suggest that larger EVs contain RNA, it is possible that smaller RNA constructs are not as efficiently stained, and we are preferentially detecting longer RNA molecules (e.g., mRNA fragments) within EVs. However, even with these possible challenges, the data clearly demonstrate the utility of the approach and capability to quantitatively assess a range of fluorescent dyes not compatible with SMLM in single EVs.

## Conclusions

In this study, the SEVEN protocol and rapid eSRRF imaging were combined with new analysis tools to characterize count, size, and detected molecular TSPAN count of single EVs. This novel SEVEN-UP approach is compatible with a wide range of fluorescence microscopy systems (as demonstrated here for TIRF and WF microscopes) and fluorophores. In cell line model, we further demonstrated the utility of this approach by characterizing RNA-positive EVs. We expect that SEVEN-UP could accelerate research and provide important functional insights; broaden research scope and collaborations (integration of EV work with other fields); provide cost-effective methodology (easily adopted by laboratories without specialized equipment or expertise); support translational EV applications (biomarker discovery and drug delivery); and promote training and education through its accessible format.

## Supporting Information Available

Additional data processing/analysis details (Supplemental Methods) and figures (Supplemental Figures) (PDF)
Developed codes related to this work along with test images for code execution(ZIP)
