# Supplementary material for: Single Extracellular VEsicle Nanoscopy-Universal Protocol (SEVEN-UP): Accessible Imaging Platform for Quantitative Characterization of Single Extracellular Vesicles
Source: Anal Chem. 2025 Jan 13;97(3):1654–64. doi: 10.1021/acs.analchem.4c04614 (PMC11780574; doi:10.1021/acs.analchem.4c04614)
Supplement: Supplementary file 1 — ac4c04614_si_001.pdf [file ac4c04614_si_001.pdf]

Supporting Information

Single Extracellular VEsicle Nanoscopy-Universal Protocol (SEVEN-UP): accessible imaging platform for quantitative characterization of single extracellular vesicles

Andras Saftics[1], Benjamin Purnell[1], Balint Beres[1,2], S. Thompson[1], Nan Jiang[1], Ima Ghaeli[1], Carinna Lima[1], Brian Armstrong[3], Kendall Van Keuren-Jensen[4], Tijana Jovanovic-Talisman[1]*

[1] Department of Cancer Biology and Molecular Medicine, Beckman Research Institute, City of Hope Comprehensive Cancer Center, Duarte, CA 91010, USA
[2] Department of Automation and Applied Informatics, Faculty of Electrical Engineering and Informatics, Budapest University of Technology and Economics, Budapest, H-1111, Hungary
[3] Light Microscopy/Digital Imaging Core, City of Hope Comprehensive Cancer Center, Duarte, CA 91010, USA
[4] Neurogenomics Division, Translational Genomics Research Institute, Phoenix, AZ 85004, USA

***Corresponding author:** Tijana Jovanovic-Talisman, E-mail: ttalisman@coh.org

Table of Content

S1 Supplemental Methods

S1.1 Antibodies

S1.2 Isolation of HEK 293T EVs and staining with SYTO RNASelect dye

S1.3 Image acquisition

S1.4 Image reconstruction

S1.5 Image alignment

S1.6 Localization precision and resolution assessment of SMLM images

S1.7 Resolution and signal-to-background ratio assessment of $SRRF^{TIRF}$ and $SRRF^{WF}$ images

S1.8 Voronoi tessellation-based clustering of SMLM localizations

S1.9 Correlative $SRRF^{TIRF}$-SMLM image analysis (640-nm channel)

S1.10 Colocalization analysis of 488/640-nm channel $SRRF^{TIRF}$ and 475/630-nm channel $SRRF^{WF}$ images

S1.11 Calibration of $SRRF^{TIRF}$ and $SRRF^{WF}$ signal using rEVs as reference sample

S1.12 Statistical information and graphics

S2 Supplemental Figures

Figure S1. $SRRF^{TIRF}$ image reconstruction process using the eSRRF method.

Figure S2. Localization precision, resolution, and signal-to-background ratio.

Figure S3. Determination of thresholding function to minimize over-segmentation of initial segments with low variation in intensity.

Figure S4. EV surface density per ROI of pEVs detected by $SRRF^{WF}$ and $SRRF^{TIRF}$ imaging performed in dSTORM imaging buffer or in PBS.

Figure S5. Confusion matrix for evaluating the segmentation accuracy of initially identified $SRRF^{TIRF}$ segments in $SRRF^{TIRF}$-SMLM test images acquired on pEVs.

Figure S6. Correlation of $SRRF^{TIRF}$- and SMLM-determined size and molecular TSPAN content of individual pEVs and rEVs in the test set.

Figure S7. Characterization of rEVs using NTA.

Figure S8. Characterization of SEC-isolated hEVs using UV-vis spectroscopy, TEM, dot blot, and NTA.

Figure S9. $SRRF^{TIRF}$-SMLM images acquired on hEVs stained with and without SYTO RNASelect dye.

S3 Supplemental References

S1 Supplemental Methods

S1.1 Antibodies

Anti-CD9 (BioLegend, Inc.; Cat# 312102; San Diego, CA, USA), anti-CD63 (Novus Biologicals, LLC; Cat# NBP2-42225; Centennial, CO, USA), and anti-CD81 (BioLegend, Inc.; Cat# 349502) antibodies (Abs) were used for microscopy. Anti-cytochrome C (BD Biosciences; Cat# 556432; Franklin Lakes, NJ, USA) and anti-syntenin (Abcam Ltd.; Cat# ab19903; Waltham, MA, USA) primary Abs as well as IRDye 800CW goat anti-mouse (LI-COR, Inc.; Cat# 926–32210; Lincoln, NE, USA) and IRDye 680RD goat anti-rabbit (LI-COR, Inc.; Cat# 926-68071) secondary Abs were used for dot blots.

S1.2 Isolation of HEK 293T EVs and staining with SYTO RNASelect dye

Cell culture. Human embryonic kidney (HEK) 293T cells (ATCC; Cat# CRL-3216; Manassas, VI, USA) were cultured in Dulbecco's modified Eagle's medium (DMEM; ATCC; Cat# 30-2002) supplemented with 10% fetal bovine serum (FBS; Avantor, Inc.; Cat# 97068-091; Radnor, PA, USA), 2 mM L-glutamine (ATCC, Cat# 30-2214), and 100 U/mL penicillin-streptomycin (MilliporeSigma; Cat# P0781-100ML; Burlington, MA, USA) at 37°C and 5% CO_2. After plating the cells in 75 cm^2 flasks, the FBS was replaced with exosome-depleted FBS (Thermo Fisher Scientific Inc.; Cat# A2720801). When the concentration reached approximately 3×10^6 cells per flask, the cells were split into 150-mm cell culture dishes supplied with 40 mL of media containing exosome-depleted FBS. After three days of incubation, when cell confluency reached 80%, the culture media was collected. To remove cell debris, the collected cell culture media was centrifuged at 300 ×g for 10 min at room temperature (RT).

Isolation of HEK 293T EVs. HEK 293T EVs (hEVs) were isolated from the collected conditioned cell culture media. Briefly, 80 mL of conditioned HEK 293T cell culture media was concentrated to ~0.4 mL using Vivaspin® 20 100 kDa centrifugal concentrator (Sartorius AG; Cat# VS2041; Göttingen, Germany) as described before[1, 2]. EVs were isolated using 70 nm qEV original Legacy size exclusion chromatography (SEC) column from Izon Science Ltd. (Cat# SP1; Christchurch, New Zealand). The column was equilibrated with phosphate buffered saline (PBS) and the first five fractions (F1-F5) containing a high concentration of hEVs and low concentration of protein impurities were combined for further experiments.

Staining of HEK 293T EVs with SYTO RNASelect. SEC-isolated hEVs were first treated with RNase A. Briefly, 1 µL of 10 mg/mL RNase A (Thermo Fisher Scientific Inc.; Cat# EN0531) was added to 100 µL HEK 293T EV sample and incubated for 30 min at 37 °C. Next, dithiothreitol (DTT; 102 mM in PBS; RPI, Corp; Cat# D11000-10.0; Mount Prospect, IL, USA) and Ambion™ RNase inhibitor (40 U/µL; Thermo Fisher Scientific Inc.; Cat# AM2682) were added to the EV sample to reach a final DTT concentration of 1 mM and final inhibitor amount of 200 U. The mixture was incubated for 5 min at 37 °C. Next, RNase A-treated EVs were stained with SYTO RNASelect dye. Briefly, 5 mM SYTO RNASelect was diluted to 1 mM stock solution in dimethyl sulfoxide (DMSO; Tocris Bioscience; Cat# 3176; Bristol, UK). The SYTO RNASelect stock solution was then added to the EV sample solution to reach a final concentration of 10 µM and the mixture was incubated for 20 min at 37 °C; in control experiments, DMSO without RNASelect was used. Subsequently, the excess dye and additional reagents were removed from 100 µL of

sample using 70 nm qEV single Legacy SEC column (Izon Science Ltd; Cat# SP2) as described elsewhere[1, 2].

S1.3 Image acquisition

While imaging, coverslips were kept in direct stochastic optical reconstruction microscopy (dSTORM) imaging buffer containing mercaptoethylamine (MEA) as well as catalase, glucose, and glucose oxidase (GLOX)[3]. To test the effect of dSTORM buffer on Super Resolution Radial Fluctuations (SRRF) image quality and number of SRRF-detected EVs, we also used phosphate buffer saline (PBS) as control imaging buffer. $SRRF^{TIRF}$ images were acquired as 500 frame sequences over 256 × 256-pixel (40.96 × 40.96 µm) region of interest (ROI) areas using the 488-nm and/or 640-nm laser lines. 488-nm channel $SRRF^{TIRF}$ images were captured with 50 ms exposure time and 1.5% laser power (~2 mW, recorded at the optical fiber outlet of the laser launch) for green fluorescent protein (GFP) and 10% laser power (~11 mW at the fiber) for SYTO RNASelect. 640-nm channel SRRF images were captured with 30 ms exposure time and 10% of laser power (~12 mW at the fiber) for Alexa Fluor 647 (AF647). Single molecule localization microscopy (SMLM) images were acquired as 25,000 frame sequences over 256 × 256-pixel (40.96 × 40.96 µm) ROI areas using 640-nm laser line; images were captured with 10 ms exposure time and 100% laser power (~125 mW at the fiber). For pooled human plasma (pEV) samples and fluorophore assessment using Surface Assay for Molecular Isolation (SAMI)[4] surfaces, 640-nm $SRRF^{TIRF}$ acquisition was followed by 640-nm SMLM acquisition. For recombinant EV (rEV) samples, 640-nm $SRRF^{TIRF}$ acquisition was followed by 640-nm SMLM acquisition, and 488-nm $SRRF^{TIRF}$ acquisition. For hEV samples, 640-nm SMLM acquisition was followed by 488-nm $SRRF^{TIRF}$ acquisition. SMLM and $SRRF^{TIRF}$ images were acquired with the NIS-Elements software (Nikon Instruments Inc.; version 5.41.02).

For $SRRF^{WF}$ image acquisition, coverslips were kept in dSTORM buffer (or PBS where indicated) during imaging. Images were acquired as 200 frame sequences over 1024 × 1024-pixel (105.65 × 105.65 µm) ROI areas. Images were captured with 150 ms exposure time and 100% illumination power in the 475-nm and 630-nm channels. The pEV samples were imaged in the 630-nm channel while rEV samples were imaged in the 630-nm channel followed by acquisition in the 475-nm channel. Images were recorded using the ZEISS ZEN 3.2 (blue edition) software (Carl Zeiss Microscopy GmbH; version 3.2.0.00009) and OME-TIF formatted images were generated using the ZEISS ZEN 3.9 Lite (blue edition) software (Carl Zeiss Microscopy GmbH; version 3.9.101.01000).

S1.4 Image reconstruction

Before eSRRF processing, the raw 1024 × 1024-pixel $SRRF^{WF}$ stack images (200 frames, 105.65 × 105.65 µm, pixel size of 103 nm) were downsized to 550 × 550-pixel stack images with cubic interpolation, using ImageJ2. Our objective was to generate $SRRF^{WF}$ images that had a similar pixel size to $SRRF^{TIRF}$ images. We noticed that higher quality $SRRF^{WF}$ images could be reconstructed when image downsizing was applied prior to eSRRF processing instead of applying lower magnification in eSRRF processing. The generated single 3300 × 3300-pixel $SRRF^{WF}$ image was cropped by selecting the central 1650 × 1650-pixel (52.83 × 52.83 µm; pixel size of 32.0 nm) area for further analysis. The size of the cropped region was optimized to exclude margin areas with reduced illumination uniformity while keeping a large number of imaged EVs for analysis.

eSRRF image processing was performed in ImageJ2 (Fiji; version 1.53t) using the NanoJ-eSRRF plugin (eSRRF v1.1.0). For eSRRF processing we used the following settings: magnification of 5 ($SRRF^{TIRF}$) or 6 ($SRRF^{WF}$), sensitivity of 5 (640-nm TIRF or 630-nm WF channel) or 7 (488-nm TIRF or 475-nm WF channel), radius of 2.5 pixels, temporal average projection (AVG) as reconstruction method; vibration correction was applied except in the case of SAMI surfaces (vibration correction produced some artifacts in low-signal SAMI surfaces); selection of frame window size was set to the auto method. From the raw 256 × 256-pixel $SRRF^{TIRF}$ stack images (500 frames, 40.96 × 40.96 µm, pixel size of 160 nm) the eSRRF processing resulted in a single 1280 × 1280-pixel (40.96 × 40.96 µm; pixel size of 32.0 nm) $SRRF^{TIRF}$ image which was subsequently analyzed. 1024 × 1024-pixel $SRRF^{WF}$ stack images (200 frames, 105.65 × 105.65 µm, pixel size of 103 nm) the eSRRF processing with additional pre- and post-processing resulted in a single 1650 × 1650-pixel (52.83 × 52.83 µm; pixel size of 32.0 nm) $SRRF^{WF}$ image for subsequent analysis.

S1.5 Image alignment

SMLM images acquired in the 640-nm channel and (if applicable) SRRF images acquired in the 488-nm channel were aligned to corresponding 640-nm channel SRRF images to correct for any lateral shift between images captured in different acquisitions or in separate channels (i.e., 640-nm SRRF was always used as the reference channel in the alignment process). We generated a uniform localization grid over the foreground pixels of binarized SRRF images, and a merged coordinate-based image containing 1) 640-nm SRRF and 640-nm SMLM or 2) 640-nm SRRF and 488-nm SRRF images was generated using our custom code in MATLAB (MathWorks, Inc.; version R2023a; Natick, MA, USA). The generated coordinate-based images were aligned using the Uniform Dual Channel Alignment tool of the Nanometrix software (Nanometrix Ltd.; version 1.0.0.1; Oxford, UK; https://nanometrix.bio). The alignment process involved a density-based spatial clustering of applications with noise (DBSCAN)-based clustering of localizations in both the alignment and reference channels. DBSCAN was performed using a search radius of 50 nm with minimum points per cluster of 20, and alignment translation was conducted up to 5 iterations. Clusters matched in two channels were annotated and the centroids of detected clusters were determined. Next, an iterative centroid matching was performed in the alignment channel minimizing the distance between the corresponding cluster centroids. The alignment channel images were translated according to the resulting single x and y coordinate shift values performing a uniform alignment.

S1.6 Localization precision and resolution assessment of SMLM images

The localization precision of SMLM images was estimated based on 1) photon counts using the NIS-Elements software (Nikon Instruments Inc.); and 2) nearest neighbor analysis (NeNA)[5] of localizations using MATLAB codes published by Martens *et al.*[6]. The resolution of SMLM images was estimated based on Fourier ring correlation (FRC) analysis using the published MATLAB codes of Martens *et al.*[6]. The localization list of each coordinate-based SMLM image was randomly split into two subsets from which two pixel-based images were reconstructed using 10 nm pixel size. The FRC analysis was subsequently performed on the two generated images.

S1.7 Resolution and signal-to-background ratio assessment of SRRF^{TIRF} and SRRF^{WF} images

To estimate the resolution of SRRF^{TIRF} and SRRF^{WF} images we performed FRC analysis using the NanoJ-SQUIRREL ImageJ2 (Fiji) plugin[7]. The raw multiframe image stacks were randomly split into two image stack subsets on which eSRRF processing was performed as described in the ***Image reconstruction*** section **S1.4** (pre-processing of SRRF^{WF} images prior to eSRRF processing was also performed according to the detailed protocol). FRC resolution map was calculated from the two SRRF images generated from the two image stack subsets in NanoJ-SQUIRREL, using a block size per axis of 20.

We determined a signal-to-background ratio (SBR) value corresponding to each SRRF^{TIRF} and SRRF^{WF} image of pEVs and rEVs. We calculated the SBR per ROI as the ratio of the mean intensity value of ROI foreground signal relative to the mean intensity value of the ROI background signal. The foreground and background region of each image were determined using the binary image as a mask generated in the initial segmentation step of the SRRF image analysis.

S1.8 Voronoi tessellation-based clustering of SMLM localizations

SMLM localizations were clustered using our MATLAB code utilizing a Voronoi tessellation-based clustering algorithm of Andronov *et al.*[8] with modifications for EV detection[9]. For detecting small EVs in pEV and rEV samples, a maximum Voronoi cell area of 450 nm^2, minimum number of localizations / cluster of 25, maximum number of localizations / cluster of 5,000, minimum cluster diameter of 30 nm, and maximum cluster diameter of 400 nm were applied; for detecting large EVs, a maximum Voronoi cell area of 600 nm^2, minimum number of localizations / cluster of 500, minimum cluster diameter of 400 nm, and maximum cluster diameter of 6,000 nm were applied; molecule counts were calculated using an average number of localizations per fluorescent reporter (α) of 10, determined from SAMI experiments[4]. For detecting small EVs in hEV samples, a maximum Voronoi cell area of 450 nm^2, minimum number of localizations / cluster of 42, maximum number of localizations / cluster of 15,000, minimum cluster diameter of 30 nm, and maximum cluster diameter of 400 nm were applied; for detecting large EVs, a maximum Voronoi cell area of 600 nm^2, minimum number of localizations / cluster of 500, minimum cluster diameter of 400 nm, maximum cluster diameter of 6,000 nm were applied; molecule counts were calculated using an α of 28, determined from SAMI experiments in conditions without fixation.

In the case of clusters detected as small EVs, localizations that were surrounding the initially detected cluster boundary but were not part of the cluster (characteristic of Voronoi tessellation-based clustering), were assigned to the clusters. Localizations spatially outlying from the clusters were excluded using the *isoutlier* built-in MATLAB function. Localizations were determined as outliers if their coordinates were more than 3.5 of scaled median absolute deviations away from the median of cluster localizations. EV diameter was defined as the diameter of an equivalent circle possessing the area of the detected SMLM segment.

S1.9 Correlative SRRF^{TIRF}-SMLM image analysis (640-nm channel)

Here, details are provided as a supplementary of section ***Correlative SRRF^{TIRF}-SMLM image analysis (640-nm channel)*** of the main text. The label of each step of the analysis procedure corresponds to the same label of the related section in the main text.

a. Image binarization and pre-processing. First, eSRRF-processed $SRRF^{TIRF}$ images were normalized and binarized using either an optimized intensity threshold (determined as 0.35% of maximum image intensity for 640-nm $SRRF^{TIRF}$ images in the case of pEV and rEV samples) or using an adaptive thresholding method (SAMI surfaces). For adaptive thresholding of images acquired on SAMI surfaces we used MATLAB's built-in *adaptthresh* function using a sensitivity parameter of 0. Here, local threshold values were determined using Gaussian weighted mean in the neighborhood. After thresholding, the foreground binary pixels were labeled by connected component labeling with equivalence class resolution (using the *bwlabel* function and 8-connected neighborhoods). Next, a 7-pixel wide frame region was cropped from the image to avoid artifacts typical in the edge of SRRF images. To limit the number of binary segments affected by the cropping (i.e., those which were significantly cut), segments detected in a 10-pixel wide image frame region with an eccentricity larger than 0.70 or circularity smaller than 0.85 were eliminated (a perfect circle has an eccentricity of 0 and circularity of 1). In addition, segments with an aggregate area smaller than 40 pixels and segments which had one-dimensional bounding box were not processed in subsequent segmentation steps.

b. Segment image transformation. Each labelled segment (termed as initial segment) was cropped out over the bounding box region and was processed individually. The initial segment intensity could encompass the signal of multiple EV components. Our goal was to determine the number of underlying components of each initial segment. The segment image intensity (i) was cubic root transformed ($i^{1/3}$) and the Laplacian (second derivative) of $i^{1/3}$ was calculated ($\nabla^2 i^{1/3}$) to improve peak separation. The generated $\nabla^2 i^{1/3}$ image segments were smoothed using median and Gaussian filtering, to account for the sensitivity of Laplacian image to intensity roughness in the case of images with reduced signal level. Filter size was set to 4 by 4 pixels for median filtering and 3 by 3 pixels for Gaussian filtering.

c. Peak detection. Peak detection was performed both on the normalized $i^{1/3}$ and normalized $\nabla^2 i^{1/3}$ segments using the *peaks2* MATLAB function developed by Tikuišis[10]. For $i^{1/3}$ image segments, peaks were detected at a minimum normalized intensity threshold of 0.4; for $\nabla^2 i^{1/3}$ segments, a minimum normalized intensity of 0.2 and a minimum distance between peaks of 3 pixels was set as thresholds of peak detection. The number of segment components was determined based on the number of detected $\nabla^2 i^{1/3}$ or $i^{1/3}$ peaks ($N_p[\nabla^2 i^{1/3}]$ and $N_p[i^{1/3}]$, respectively), in case of $N_p[\nabla^2 i^{1/3}] > 1$ or $N_p[i^{1/3}] > 1$. We found that if the number of all detectable segment peaks without thresholding, $N_{p,total}[\nabla^2 i^{1/3}]$, was large (i.e., the $\nabla^2 i^{1/3}$ signal had a high noise), the $\nabla^2 i^{1/3}$-based peak detection had an increased tendency of over-segmentation. Up to $N_{p,total}[\nabla^2 i^{1/3}] = 6$, $N_p[\nabla^2 i^{1/3}]$ was considered as the number of segment components, while in the case of $N_{p,total}[\nabla^2 i^{1/3}] \geq 7$ the number of components was defined as $N_p[i^{1/3}]$. In case only a single peak was detected, a machine learning-based ordinal classification model, which was trained using the SMLM clusters as ground truth, was performed to decide whether multiple EVs could be detected within that segment.

d. Feature extraction. Morphological features (eccentricity, circularity, solidity, and extent), coefficient of variation of object intensity, as well as histogram of oriented gradients (HOG) features were extracted for individual initial segments. For HOG feature extraction, each initial segment image was resized into a 30 × 30-pixel image using bicubic interpolation. The number of HOG features was reduced from 144 to 6 performing principal component analysis (PCA), where the selected first 6 principal components represented 60% of the total variance in the HOG feature space. In total, 11 features were considered for regression analysis. To avoid the over-segmentation of initial segments which had a low coefficient of variation (CV) in intensity and low eccentricity (E) in morphology, segments obeying the threshold function of (–

$0.67 \times CV^3 + 0.16 \times CV^2 - 0.14 \times CV + 1.03) > E$ were not processed in further segmentation. The function was determined to fit to the ~80% confidence region boundary of the distribution of multi-EV segments (limiting the number of thresholded multi-EV segments while thresholding out most low intensity variation segments), **Fig. S3**.

e. SRRF^{TIRF}-SMLM colocalization of initial segments. To evaluate segmentation and train a regression model to predict EV size and molecular content based on SRRF, clustered SMLM images were used as the ground truth. For training and validation, the initial SRRF^{TIRF} image segments (i.e., initial SRRF^{TIRF}-detected EVs) were annotated with their true number of components using the SMLM clusters. The segmented SRRF^{TIRF} images were overlayed with clustered SMLM images; overlaps between SRRF^{TIRF} segments and SMLM clusters were evaluated. SRRF^{TIRF}-SMLM colocalizations were identified if at least 10% of the SMLM cluster area was overlapping with the SRRF^{TIRF} segment. In case an SMLM cluster was found overlapping with multiple SRRF^{TIRF} segments, colocalization was considered only for the largest overlapping pairs, given that at least 80% of SMLM cluster area was in overlap with the selected SRRF^{TIRF} segment (making sure that an SMLM cluster was assigned to the corresponding SRRF^{TIRF} segment with high confidence).

f. Training classification model. Using the extracted initial segment features and annotated class labels (number of components), a medium Gaussian support vector machine (SVM) model with Gaussian kernel was trained to enhance the segmentation of multipeak objects identified as single peaks in the peak detection step. Due to the small number of training set segments which had more than three overlapping SMLM clusters, the model was trained to classify segments into classes representing 1-, 2-, or 3-component segments. The training was performed using MATLAB's Classification Learner app. The kernel size of the medium Gaussian SVM model was 3.3, determined automatically in the software. The model was selected from a pool of tested classification models (apart from SVM, decision tree, k-nearest neighbors (KNN), and naive Bayes classifiers were tested) based on best classification accuracy.

g. Segmentation based on number of identified components. The pixel-based multipeak initial SRRF^{TIRF} segments were converted to localization-based image segments by generating a grid of localizations over the foreground pixels of the multipeak segments. Here, the grid density of localizations was linearly scaled according to pixel intensity in the normalized $\nabla^2 i^{1/3}$ segment image. Based on the identified number of components, the final segmentation of multipeak segments was generated using k-means clustering of grid localizations with city block (Manhattan) distance metric, which had superior performance compared to other metrics in defining separation boundary between the clusters. The density of the localization grid generated over the foreground pixels of initial SRRF^{TIRF} segments was optimized considering segmentation speed and multipeak separation performance. According to our optimized grid density, the normalized grid spacing per pixel (s) was determined using the following equation: $s = -0.1875 \times \nabla^2 i^{1/3} + 0.25$ (i.e., the spacing was 1/4th of pixel size at $\nabla^2 i^{1/3} = 0$ and 1/16th of pixel size at maximum normalized intensity, $\nabla^2 i^{1/3} = 1$). Based on the identified number of components, the final segmentation of multipeak segments was generated using k-means clustering of grid localizations with city block distance metric.

h. SRRF^{TIRF}-SMLM colocalization of final segments and correlation analysis. The overlap of SMLM clusters with the final SRRF^{TIRF} segments was evaluated in a second colocalization analysis step using the same settings as in the first one. For evaluating the correlation of SRRF^{TIRF} segment characteristics with EV size and molecular TSPAN content, we selected those final SRRF^{TIRF} segments in the training set for which an overlapping single SMLM cluster was identified and the number of SMLM-detected TSPAN molecules per EV was smaller or equal to 200 (SMLM

clusters with very high molecule count were not considered in regression due to reduced segmentation accuracy of corresponding large multipeak SRRF blobs). Using a linear regression model, we correlated the diameter of $SRRF^{TIRF}$ segments (D_S^{SRRF}) with the diameter of corresponding SMLM clusters (D_{EV}^{SMLM}) as well as the square root of the integral of normalized intensity over the $SRRF^{TIRF}$ segment (I_S^{SRRF}) with the number of detected TSPAN molecules within SMLM clusters (TSPAN count per EV, M_{EV}^{SMLM}). $SRRF^{TIRF}$ segment diameter was defined as the diameter of a circle with an area equivalent to the area of a $SRRF^{TIRF}$ segment.

i. Validation of EV detection and segmentation. Segmentation performance was evaluated on the test data set. The developed method including **steps a-d** and **g** was performed, and the number of segmented components of each initial segment was compared with the annotated number of components of that initial segment obtained from **step e** as the number of overlapping SMLM clusters.

S1.10 Colocalization analysis of 488/640-nm channel $SRRF^{TIRF}$ and 475/630-nm channel $SRRF^{WF}$ images

488-nm $SRRF^{TIRF}$ and 475-nm $SRRF^{WF}$ images of rEVs were binarized using an intensity threshold of 0.1% and 0.8%, respectively. 488-nm $SRRF^{TIRF}$ images of hEVs were binarized using an intensity threshold of 0.35%. Threshold values were optimized to minimize the 488-nm or 475-nm channel background signal which was determined as signal without colocalized 640-nm ($SRRF^{TIRF}$) or 630-nm ($SRRF^{WF}$) channel signal. Due to the detected highly saturated signal of rEVs in 488-nm $SRRF^{TIRF}$ images, we applied a segment dilation operation on the binary images using disk structuring element with a radius of 6 pixels to fill any holes in the identified segments. Segmentation of SRRF images was performed according to **step a** in the section **S1.9** and colocalization of detected 488/640-nm channel $SRRF^{TIRF}$ segments as well as 475/630-nm channel $SRRF^{WF}$ segments was performed according to **step e**, except that colocalization was considered if at least 2% of SMLM cluster area was in overlap with the selected $SRRF^{TIRF}$ segment.

S1.11 Calibration of $SRRF^{TIRF}$ and $SRRF^{WF}$ signal using rEVs as reference sample

The mean segment diameter per ROI ($\langle D_S^{SRRF}\rangle$) and square root of mean segment intensity integral per ROI ($\langle I_S^{SRRF}\rangle$) features determined from 640 nm $SRRF^{TIRF}$ and 630 nm $SRRF^{WF}$ images were calibrated based on pre-determined reference values of a standard sample to calculate the mean EV diameter per ROI ($\langle D_{EV}^{SRRF}\rangle$) and detected mean TSPAN count per EV per ROI ($\langle M_{EV}^{SRRF}\rangle$). Here, the SMLM-determined $\langle D_{EV}\rangle$ and $\langle M_{EV}\rangle$ data of rEVs ($\langle D_{EV}^{SMLM,rEV}\rangle$ and $\langle M_{EV}^{SMLM,rEV}\rangle$, respectively) were used as reference to calibrate the SRRF-based data performing 5-fold cross-validation. First, both the $SRRF^{TIRF}$ and $SRRF^{WF}$ ROI sets acquired on rEV samples were randomly divided into training and test sets five times with 80-20% ratios. Next, in the training set, $\langle D_S^{SRRF}\rangle$ and $\langle I_S^{SRRF}\rangle$ were divided by $\langle D_{EV}^{SMLM,rEV}\rangle$ and $\langle M_{EV}^{SMLM,rEV}\rangle$, respectively, to calculate calibration sensitivities (slope of calibration curves; $a_D = \langle D_S^{SRRF,train}\rangle / \langle D_{EV}^{SMLM,rEV}\rangle$; $a_M = \langle I_S^{SRRF,train}\rangle / \langle M_{EV}^{SMLM,rEV}\rangle$). The determined calibration sensitivity values were subsequently used to calculate D_{EV}^{SRRF} and M_{EV}^{SRRF} in the test set ($\langle D_{EV}^{SRRF}\rangle = \langle D_S^{SRRF,test}\rangle / a_D$; $\langle M_{EV}^{SRRF}\rangle = \langle I_S^{SRRF,test}\rangle / a_M$). In addition, universal $SRRF^{TIRF}$-based a_D and a_M calibration sensitivities were calculated based on the rEV training set. Using this universal calibration and the above formulas, D_{EV}^{SRRF} and M_{EV}^{SRRF} were calculated for pEVs in the pEV test ROI set.

S1.12 Statistical information and graphics

Mean, standard error of the mean (SEM) and coefficient of variation (CV) values related to the presented data were calculated in GraphPad Prism. For the statistical analysis of two data sets, statistical significance of the difference between data sets was determined using two-tailed Welch's test. For statistical analysis of more than two data sets, Brown-Forsythe and Welch analysis of variance (ANOVA) tests were performed using pairwise comparisons with SMLM data as a control set and Dunnett T3 test was performed to correct for multiple comparisons. Significance levels were determined based on the resulting p-values indicated as ns (no significance) if $p \geq 0.05$, * if $p < 0.05$, ** if $p < 0.01$, *** if $p < 0.001$, and **** if $p < 0.0001$. Data files were inspected and processed in Excel (Microsoft; version 2401; Redmond, WA, USA). The presented images were generated in ImageJ2 and MATLAB. Graphs were generated in GraphPad Prism 10. Table of Contents Graphic was created in part with BioRender.com. Figures were assembled in Adobe Illustrator 2023 (Adobe Inc.; version 27.5; San Jose, CA, USA).

S2 Supplemental Figures

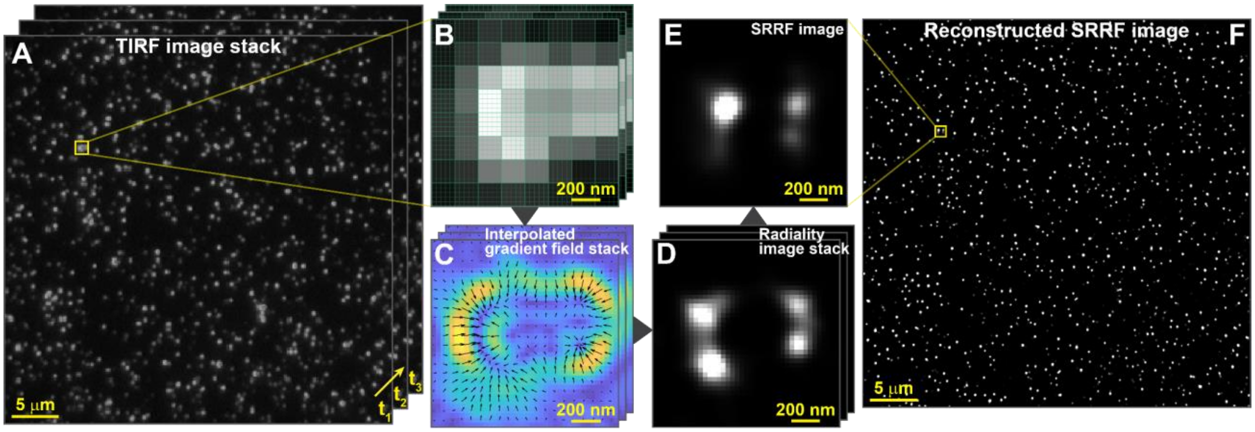

Figure S1 | SRRF^{TIRF} image reconstruction process using the eSRRF method.
The figure shows the distinct steps performed when images are processed using the enhanced SRRF (eSRRF) protocol[11]. Images acquired in total internal reflection (TIRF) illumination mode as a sequence in time *t* (TIRF image stack, **A**) were magnified using fast Hartley-transformed-based interpolation over the generated grid shown in image **B**. Next, the gradient of interpolated images was calculated (**C**); the gradient images were used to subsequently calculate a radiality image stack (**D**). Using an averaging-based projection method, a final SRRF image was reconstructed (**E**, **F**). The same principle works for widefield illumination mode.

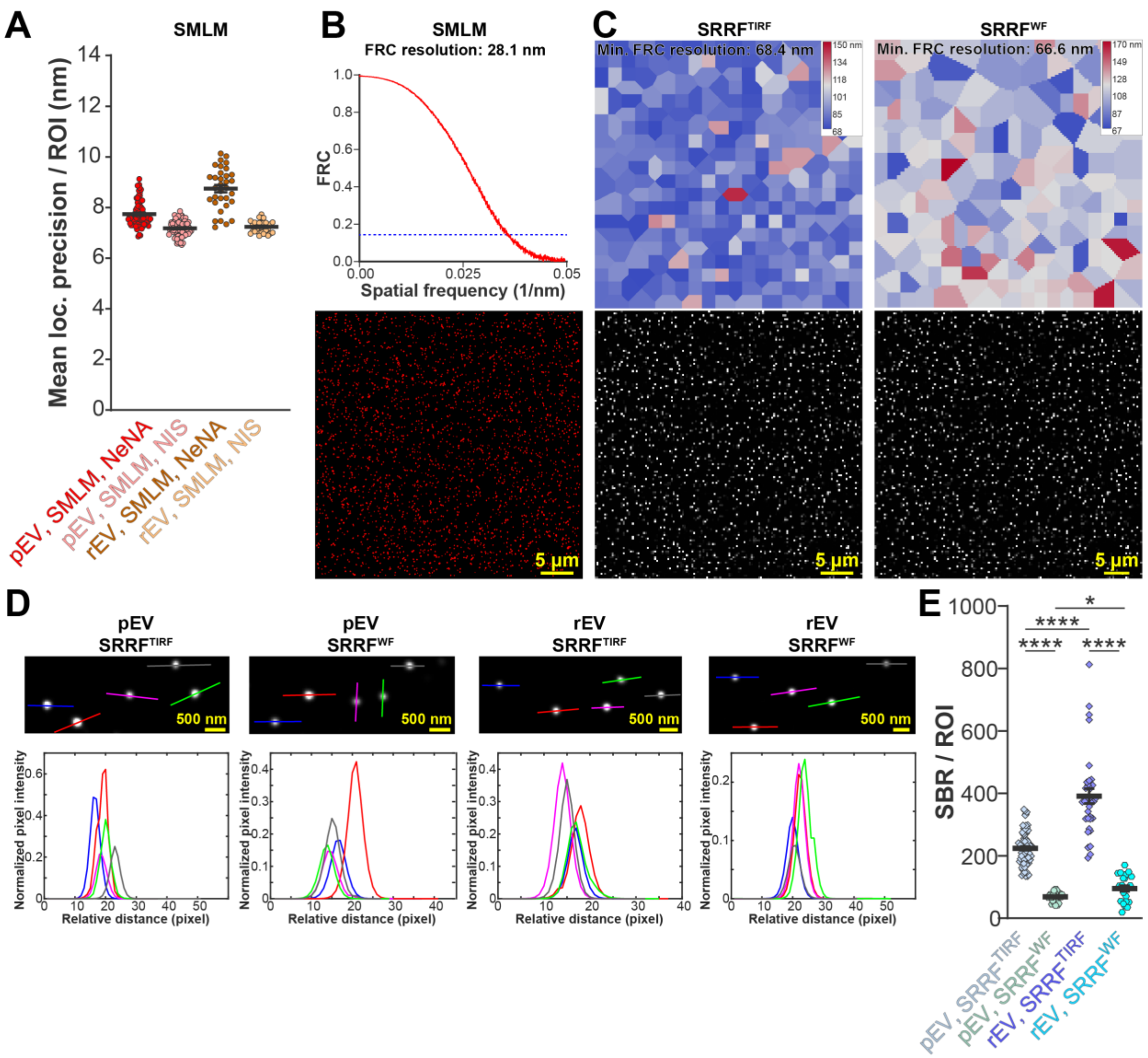

Figure S2 | Localization precision, resolution, and signal-to-background ratio.

A. Estimated mean localization precision per ROI for SMLM images acquired on pEVs (*n* = 10 independent measurements, 77 ROIs) and rEVs (*n* = 4 independent measurements, 33 ROIs). Localization precision was determined both using the NeNA method and the NIS-Elements software. Error bars represent mean ± SEM. **B.** Estimation of SMLM image resolution based on the FRC curve (top) determined for a high density SMLM image (bottom) acquired for pEV sample. The global image resolution was determined as the spatial frequency where the FRC curve crosses the 1/7 FRC value (28.1 nm). **C.** Left, estimation of $SRRF^{TIRF}$ image resolution based on the FRC map (top) determined for a high density $SRRF^{TIRF}$ image (bottom) acquired for pEV sample. The minimum resolution was determined as 68.4 nm. Right, estimation of $SRRF^{WF}$ image resolution based on the FRC map (top) determined for a high density $SRRF^{TIRF}$ image (bottom) acquired for pEV sample. The minimum resolution was determined as 66.6 nm. **D.** Representative regions from $SRRF^{TIRF}$ and $SRRF^{WF}$ images of pEVs (left) and rEVs (right) with corresponding normalized intensity profiles of EV signals recorded along the colored lines indicated in the images. **E.** Signal-to-background ratio (SBR) per ROI was quantified for pEVs and rEVs using both the training and test set (pEVs, $SRRF^{TIRF}$: *n* = 10 independent measurements, 77 ROIs; pEVs, $SRRF^{WF}$: *n* = 4 independent measurements, 27 ROIs; rEVs, $SRRF^{TIRF}$: *n* = 4 independent measurements, 33 ROIs; rEVs, $SRRF^{WF}$: *n* = 4 independent measurements, 22 ROIs).

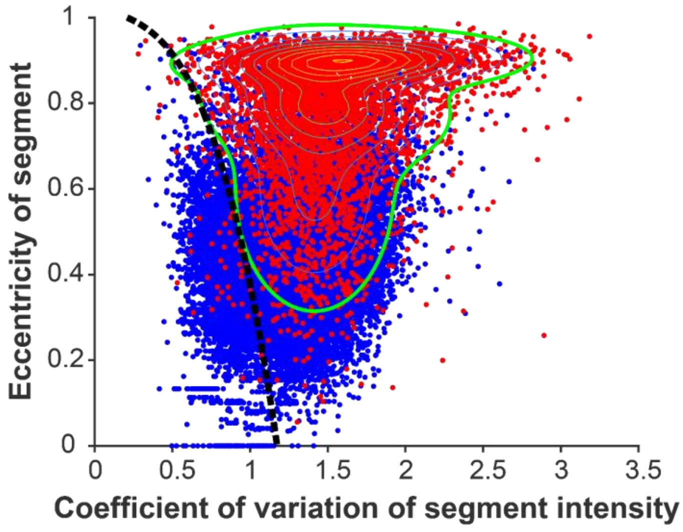

Figure S3 | Determination of thresholding function to minimize over-segmentation of initial segments with low variation in intensity.
For each detected initial segment in the pEV training set, the eccentricity (E) was plotted against the coefficient of variation (CV) of the intensity. In the generated scatter plot, the blue dots represent segments with one overlapping SMLM cluster while the red dots represent segments with multiple overlapping SMLM clusters (i.e., EVs). Segments with low intensity variation, i.e., low intensity CV had a high tendency of over-segmentation due to their sensitivity to having multiple peaks detected in their Laplacian image. Our goal was to filter out those segments which had low CV values but consider their distribution as a function of eccentricity to limit the number of thresholded segments which had multiple overlapping EVs. The distribution was fitted with a 3-component Gaussian mixture model represented by the shown contour lines; the green curve shows the 80% confidence region. To determine a thresholding function, we considered eliminating segments with low CV values while following the ~80% confidence region boundary to keep most multi-EV segments in further processing. As a result, we fitted the black dashed curve representing the 3rd order polynomial thresholding function of $-0.67\times CV^3 + 0.16\times CV^2 - 0.14\times CV + 1.03 - E = 0$. Segments on the left-hand side of this function represented by the black dashed line were not processed in further segmentation.

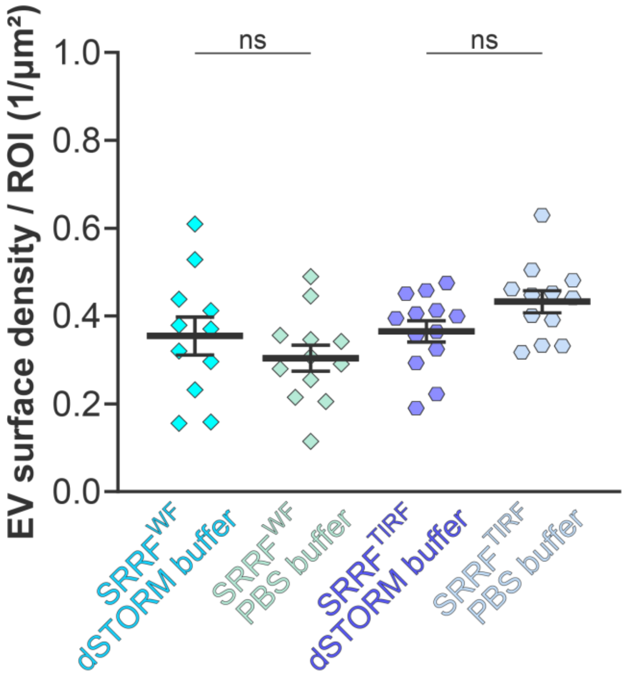

Figure S4 | EV surface density per ROI of pEVs detected by SRRF^{WF} and SRRF^{TIRF} imaging performed in dSTORM imaging buffer or in PBS.

Comparison of EV surface density per ROI values determined from 630 nm channel SRRF^{WF} and 640 nm channel SRRF^{TIRF} images of pEVs acquired while coverslips were imaged either in dSTORM or PBS buffer.

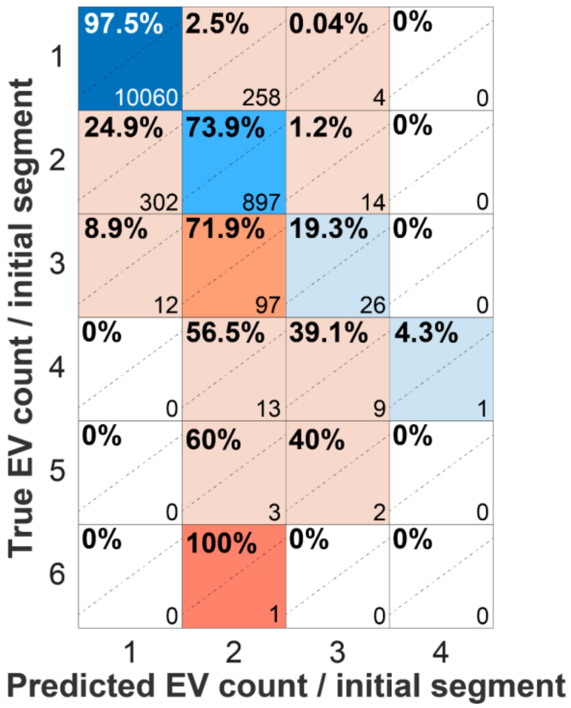

Figure S5 | Confusion matrix for evaluating the segmentation accuracy of initially identified SRRF^{TIRF} segments in SRRF^{TIRF}-SMLM test images acquired on pEVs.
SRRF^{TIRF} segmentation accuracy was assessed by comparing the number of SMLM-detected EVs overlapping with the identified initial SRRF^{TIRF} segments (true EV count / initial segment) and the number of components of initial SRRF^{TIRF} segments determined in our trained segmentation method (predicted EV count / initial segment). The percentage value in the top left corner of each cell represents the percentage of segments in the specific cell relative to the entire true class; the value in the bottom right corner of each cell represents the total number of segments in the specific cell. n = 4 independent measurements, 19 ROIs. (Of note, only initial SRRF^{TIRF} segments with overlapping SMLM-detected EVs were counted in the confusion chart.)

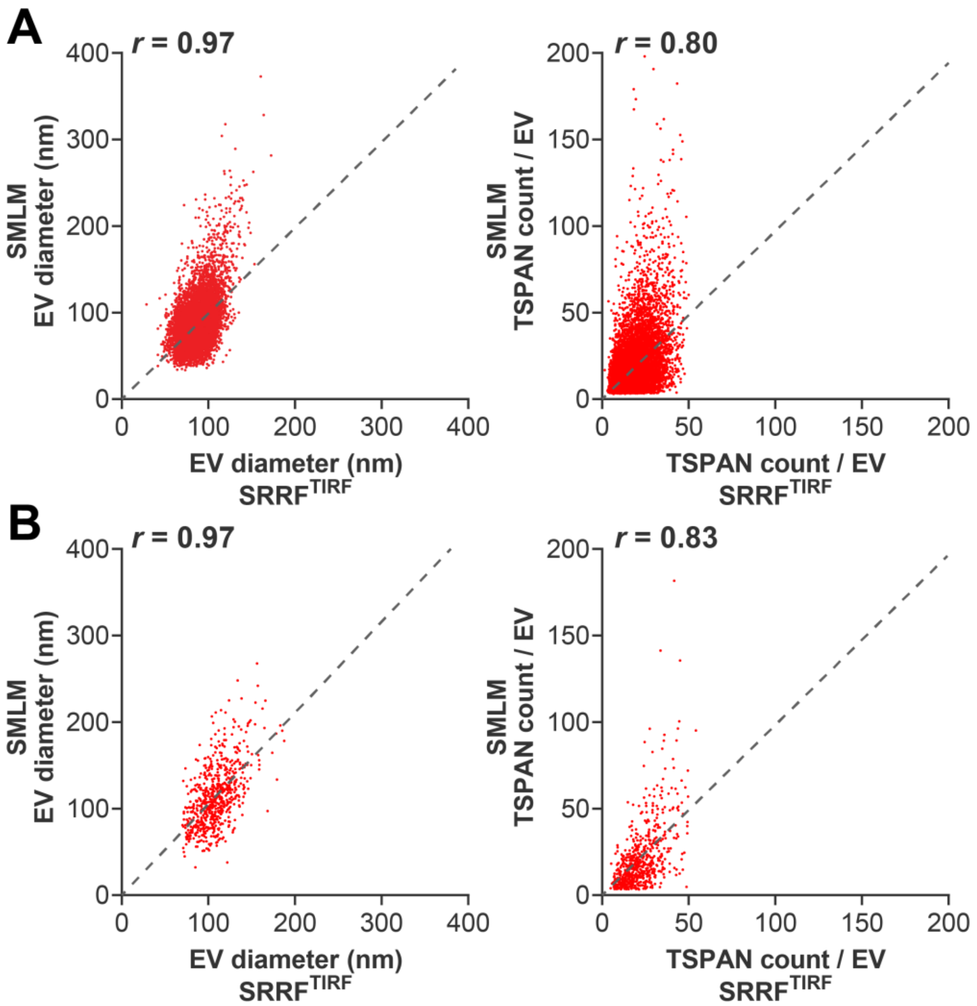

Figure S6 | Correlation of SRRF^{TIRF}- and SMLM-determined size and molecular TSPAN content of individual pEVs and rEVs in the test set.

A. Correlated SRRF^{TIRF}-based diameter and SMLM-based diameter (left) as well as correlated SRRF^{TIRF}-based TSPAN count and SMLM-based TSPAN count (right) of individual pEVs colocalized in the overlayed SRRF^{TIRF} and SMLM images of the test set (n = 10 independent measurements, 19 ROIs). The SRRF^{TIRF}-based diameter and TSPAN count per EV were calculated according to the calibrations determined in Fig. 2C and Fig. 2D, respectively. **B.** Correlated SRRF^{TIRF}-based diameter and SMLM-based diameter (left) as well as correlated SRRF^{TIRF}-based TSPAN count and SMLM-based TSPAN count (right) of individual rEVs colocalized in the overlayed SRRF^{TIRF} and SMLM images of the test set (n = 4 independent measurements, 8 ROIs). The SRRF^{TIRF}-based diameter and TSPAN count per EV were calculated according to the calibrations determined in Fig. 3D and Fig. 3E, respectively. Linear regression models (gray dashed line) representing the relationship of SRRF^{TIRF} and SMLM data with corresponding correlation coefficient (r) values are shown.

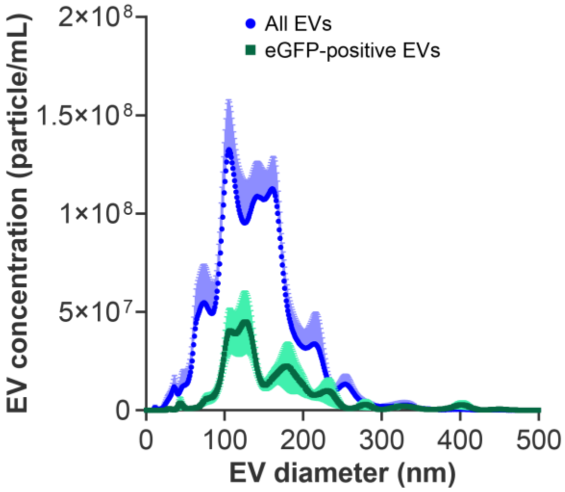

Figure S7 | Characterization of rEVs using NTA.
Size distribution of all rEVs (blue) and eGFP-positive rEVs (green) (data represent mean ± SEM, 9 runs, *n* = 3 independent measurements). The mean EV concentration and mean EV diameter with SEM were determined as $(1.34 \pm 0.06)\times10^{10}$ particle/mL and 142 ± 3 nm for all EVs and $(3.7 \pm 0.6)\times10^{9}$ particle/mL and 171 ± 16 nm for eGFP-positive EVs. The mean eGFP-positive rEV ratio was calculated as 28 ± 3%.

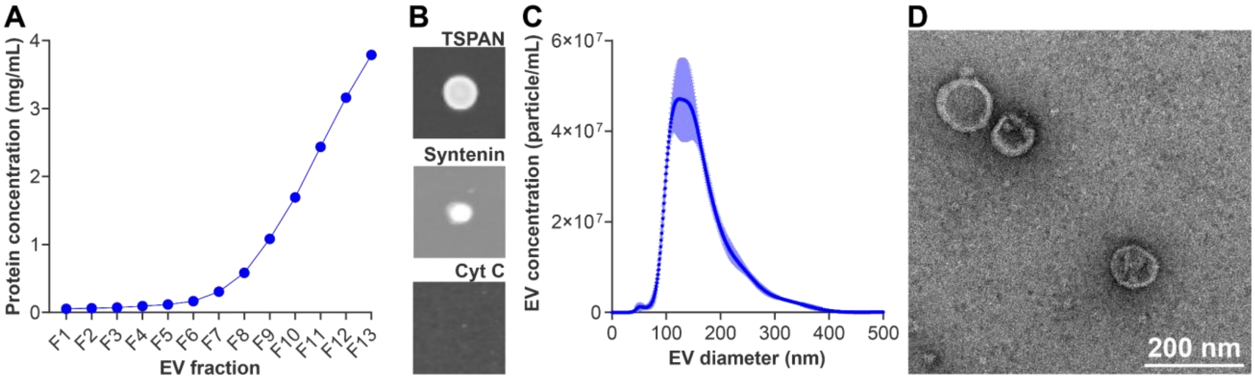

Figure S8 | Characterization of SEC-isolated hEVs using UV-vis spectroscopy, TEM, dot blot, and NTA.

A. Protein concentration of SEC-collected fractions (F1-F13) determined by UV-vis spectroscopy. Fractions 1-5 were combined (F1-F5) and used in further experiments. **B.** Dot blots of F1-F5, assessing the TSPAN (CD9, CD63, and CD81 combined), syntenin, and cytochrome C (cyt C) content. **C.** Size distribution of EVs in F1-F5 measured by NTA (data represent mean ± SEM, 3 runs). The mean EV concentration and mean EV diameter were determined as $(5.3 \pm 0.4)\times10^9$ particle/mL and 164 ± 1 nm, respectively. **D.** TEM image of EVs from F1-F5 showed intact EV morphology.

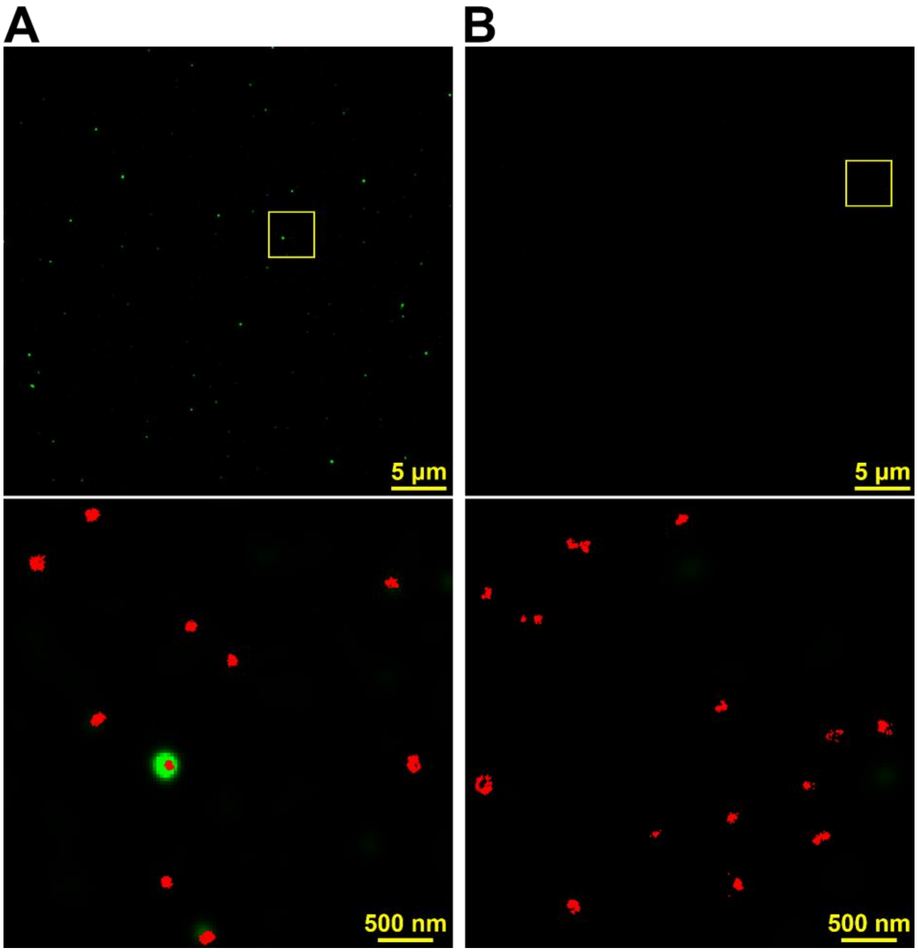

Figure S9 | SRRF^{TIRF}-SMLM images acquired on hEVs stained with and without SYTO RNASelect dye.

Representative ROIs of raw 488-nm channel SRRF^{TIRF} images acquired on hEVs stained with (**A**) and without (**B**) SYTO RNASelect dye (top). The zoomed-in images in the bottom were overlayed with their corresponding 640-nm channel SMLM localizations.

S3 Supplemental References

1. Lennon, K. M.; Wakefield, D. L.; Maddox, A. L.; Brehove, M. S.; Willner, A. N.; Garcia-Mansfield, K.; Meechoovet, B.; Reiman, R.; Hutchins, E.; Miller, M. M.; Goel, A.; Pirrotte, P.; Van Keuren-Jensen, K.; Jovanovic-Talisman, T., Single molecule characterization of individual extracellular vesicles from pancreatic cancer. *Journal of extracellular vesicles* **2019,** *8* (1), 1685634-1685634.
2. Lennon, K. M.; Saftics, A.; Abuelreich, S.; Sahu, P.; Lehmann, H. I.; Maddox, A. L.; Bagabas, R.; Januzzi, J. L.; Van Keuren-Jensen, K.; Shah, R.; Das, S.; Jovanovic-Talisman, T., Cardiac troponin T in extracellular vesicles as a novel biomarker in human cardiovascular disease. *Clin Transl Med* **2022,** *12* (8), e979.
3. Dempsey, G. T.; Vaughan, J. C.; Chen, K. H.; Bates, M.; Zhuang, X. W., Evaluation of fluorophores for optimal performance in localization-based super-resolution imaging. *Nat. Methods* **2011,** *8* (12), 1027-1036.
4. Golfetto, O.; Wakefield, D. L.; Cacao, E. E.; Avery, K. N.; Kenyon, V.; Jorand, R.; Tobin, S. J.; Biswas, S.; Gutierrez, J.; Clinton, R.; Ma, Y.; Horne, D. A.; Williams, J. C.; Jovanović-Talisman, T., A Platform To Enhance Quantitative Single Molecule Localization Microscopy. *Journal of the American Chemical Society* **2018,** *140* (40), 12785-12797.
5. Endesfelder, U.; Malkusch, S.; Fricke, F.; Heilemann, M., A simple method to estimate the average localization precision of a single-molecule localization microscopy experiment. *Histochem Cell Biol* **2014,** *141* (6), 629-38.
6. Martens, K. J. A.; Turkowyd, B.; Endesfelder, U., Raw Data to Results: A Hands-On Introduction and Overview of Computational Analysis for Single-Molecule Localization Microscopy. *Front Bioinform* **2021,** *1*, 817254.
7. Culley, S.; Albrecht, D.; Jacobs, C.; Pereira, P. M.; Leterrier, C.; Mercer, J.; Henriques, R., Quantitative mapping and minimization of super-resolution optical imaging artifacts. *Nat Methods* **2018,** *15* (4), 263-266.
8. Andronov, L.; Orlov, I.; Lutz, Y.; Vonesch, J. L.; Klaholz, B. P., ClusterViSu, a method for clustering of protein complexes by Voronoi tessellation in super-resolution microscopy. *Scientific reports* **2016,** *6*, 24084.
9. Saftics, A.; Abuelreich, S.; Romano, E.; Ghaeli, I.; Jiang, N.; Spanos, M.; Lennon, K. M.; Singh, G.; Das, S.; Van Keuren-Jensen, K.; Jovanovic-Talisman, T., Single Extracellular VEsicle Nanoscopy. *Journal of extracellular vesicles* **2023,** *12* (7), e12346.
10. Tikuišis, K. peaks2 - find peaks in 2D data without additional toolbox.
11. Laine, R. F.; Heil, H. S.; Coelho, S.; Nixon-Abell, J.; Jimenez, A.; Wiesner, T.; Martínez, D.; Galgani, T.; Régnier, L.; Stubb, A.; Follain, G.; Webster, S.; Goyette, J.; Dauphin, A.; Salles, A.; Culley, S.; Jacquemet, G.; Hajj, B.; Leterrier, C.; Henriques, R., High-fidelity 3D live-cell nanoscopy through data-driven enhanced super-resolution radial fluctuation. *Nat. Methods* **2023**.
